# Statistical modeling of trends in infant mortality after atmospheric nuclear weapons testing

**DOI:** 10.1371/journal.pone.0284482

**Published:** 2023-05-18

**Authors:** Alfred Körblein

**Affiliations:** Independent Researcher, Retired, Nuremberg, Germany; University of South Carolina, UNITED STATES

## Abstract

The global fallout from atmospheric nuclear weapons testing in the 1950s and 1960s caused by far the greatest exposure of mankind to ionizing radiation. Surprisingly few epidemiological studies of the possible health effects of atmospheric testing have been conducted. Here, long-term trends in infant mortality rates in the United States (U.S.) and five major European countries (EU5) were examined: The United Kingdom, Germany, France, Italy, and Spain. Bell-shaped deviations from a uniformly decreasing secular trend were found beginning in 1950, with maxima around 1965 in the U.S. and 1970 in EU5. From the difference between observed and predicted infant mortality rates, in the period 1950–2000, the overall increase in infant mortality rates was estimated to be 20.6 (90% CI: 18.6 to 22.9) percent in the U.S. and 14.2 (90% CI: 11.7 to 18.3) percent in EU5 which translates to 568,624 (90% CI: 522,359 to 619,705) excess infant deaths in the U.S. and 559,370 (90% CI: 469,308 to 694,589) in the combined five European countries. The results should be interpreted with caution because they rely on the assumption of a uniformly decreasing secular trend if there had been no nuclear tests, but this cannot be verified. It is concluded that atmospheric nuclear weapons testing may be responsible for the deaths of several million babies in the Northern Hemisphere.

## Introduction

**Fig 1:**
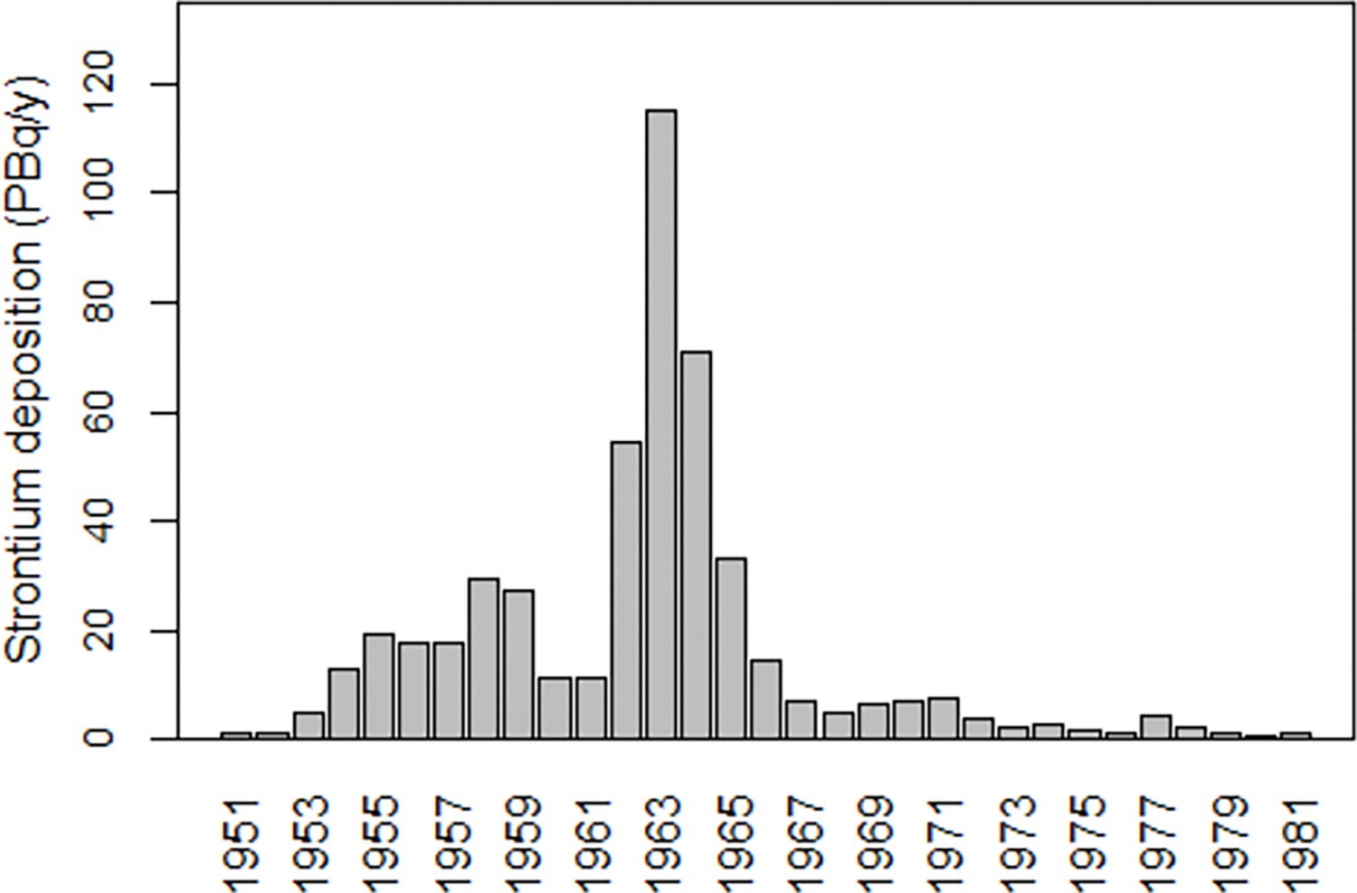
Annual strontium-90 deposition in the northern hemisphere from atmospheric nuclear testing. Created by the author from UNSCEAR 2000 [2] Annex C, Table 7.

The testing of nuclear weapons in the atmosphere resulted in worldwide exposure to radioactive fallout from the explosions. Atmospheric nuclear testing was at its height in the late 1950s and early 1960s. The cumulative explosive power of the tests corresponded to 545 megatons of 2,4,6-trinitrotoluene (TNT), which is equivalent to 40,000 atomic bombs of the type that was dropped on Hiroshima at the end of the Second World War (WW2). The collective dose to the world population was estimated at 30 million Person-Sievert (PersSv) [1] p.99), which can be compared with 600,000 PersSv from the Chernobyl accident in 1986 [1] p.115). Fig 1 shows the annual strontium deposition in the northern hemisphere from atmospheric nuclear testing. Internal exposure was greatest in the Northern Hemisphere where most of the weapons testing took place.

Epidemiological studies of possible health effects from atmospheric testing focused on childhood leukemia. Radiation-induced excess risk of childhood leukemia is apparent within some five years of exposure, so the increase in childhood leukemia after exposure to fallout is expected to appear in the late 1960s. A temporal correlation study of childhood leukemia in some Nordic countries (Denmark, Finland, Iceland, Norway, and Sweden) with the fallout from atmospheric nuclear weapons testing found little evidence of increased incidence of leukemia among children born in these years [3]. During the high exposure period, children would have been subjected to an additional equivalent dose of around 1.5 mSv, similar to doses received by children in several parts of central and eastern Europe owing to the Chernobyl accident, and about 50% greater than the annual dose equivalent to the red bone marrow of a child from natural radiation [3]. Rates of leukemia in the high exposure period were slightly higher than in the surrounding medium exposure period (relative risk for ages 0–14: 1.07, 95% confidence interval 1.00 to 1.14; for ages 0–4: 1.11, 1.00 to 1.24). A later study also found “no evidence of a wave of excess cases (of childhood leukemia incidence) corresponding to the peak of radioactive fallout from atmospheric weapons testing which was expected based on conventional risk estimates” [4].

In the late 1960s, Sternglass investigated trends in infant mortality rates in the United States following the Nevada test series and the Pacific tests in the 1950s [5]. After 1950, infant mortality rates deviated from an exponentially declining trend observed in 1935–1950 (see S1 Fig in S1 File). Citing early warnings by Sakharov [6], Sternglass attributed the excess infant mortality in the United States to immune system damage from strontium-90 in the fallout from atmospheric nuclear weapons testing. He estimated that in the U.S. “some 400,000 infants of less than one year of age probably had died as the result of nuclear fallout between 1950 and 1965” [7]. Sternglass’ claims have been questioned [8–11]; a major criticism was that "a careful evaluation of the available data does not demonstrate a direct relationship between fallout and the observed trend changes" [10].

Whyte analyzed data on first-day neonatal mortality from England and Wales and the U.S. [12]. The trends were very similar in the two countries: an upward deviation from the trend of the preceding years starting in 1950, with a broad maximum around 1965 and a decline until about 1980 when the data resumed the trend of the years 1935–1950, see S2 Fig in S1 File. For the United States, Whyte identified 280,000 excess neonatal deaths during 1951–1980 compared with the trend during the remaining years of the study period (1935–1987). Among environmental factors which might explain the excess mortality, Whyte mentions exposure to strontium-90 resulting from atmospheric weapons testing.

The present author investigated the development of perinatal mortality in West Germany in the period 1955–93 and found similar deviations from a monotonously decreasing trend, but with a maximum around 1970 [13], see S3 Fig in S1 File. As a possible explanation for the time lag between exposure and effect, he suggested that radioactive strontium 90 ingested by girls at about age 14, during the period of increased bone growth, impairs their immune systems, which in turn may lead to increased mortality of children born to these girls at later ages.

The present exploratory study adopts the approach used in [13] to examine infant mortality trends in the United States before and after atmospheric nuclear weapons testing and compares the results with those of the combined data from five European countries (United Kingdom, Germany, France, Italy, and Spain).

## Data and methods

As mentioned above, Körblein reported an association between perinatal mortality in West Germany and the calculated strontium burden of pregnant women [13]. His rather complex analysis included data on annual strontium concentration in the fallout and the maternal age distributions for each year of the study period (1955–1993). However, a nearly perfect fit to this data was also obtained with a regression model using two bell-shaped excess terms (lognormal density distributions), superimposed on a uniformly decreasing trend, see S4 Fig in S1 File. Therefore, lognormal distributions are used in the present study to model the trend of infant mortality rates. Infant mortality was chosen as the endpoint because such data are available online for many countries, which is not the case for perinatal mortality.

Infant death is defined as the death of a live-born infant within the first year. Infant mortality is the number of infant deaths per 1000 live births. Annual numbers of live births and infant deaths for the United States (U.S.) and the United Kingdom (UK), France, Italy, and Spain are provided at https://mortality.org. German infant mortality data for 1931–1943 were obtained from the German Federal Office of Statistics on request. The German data from 1946 to 2021 are published in an article available for download on their website, https://www.destatis.de [14]. Data for 1944 and 1945 do not exist.

The present analysis of infant mortality in the United States uses data from 1934 through 2018. For Europe, the pooled data from the United Kingdom (UK), Germany, France, Italy, and Spain, 1931–2018, were chosen.

Two competing mathematical models were used for the undisturbed trend: (1) a modified logistic function allowing for a lower limit of infant mortality, as in [13]; and (2) an exponential trend with a third-degree polynomial of time. The full regression Model (1) with 3 bell-shaped excess terms is non-linear and has the following analytical form:

E(y(t))=α+(1−α)/(1+1/exp(β1+β2∙t+β5/t/exp((log(t)−β6)^2/2/β7^2)+β8/t/exp((log(t)−β9)^2/2/β10^2)+β11/t/exp((log(t)−β12)^2/2/β13^2)) (1)

Here, *E*(*y*(*t*)) is the predicted value of infant mortality *y*(*t*). Time *t* is the calendar year minus 1930; the constant *α* is the lower limit of infant mortality; *β*_1_ and *β*_2_ are trend parameters; and *β*_5_ through *β*_13_ are parameters defining the three lognormal functions.

Model (2) has the following form:

E(y(t))=exp(β1+β2∙t+β3∙t2+β4∙t3+β5/t/exp((log(t)−β6)^2/2/β7^2)+β8/t/exp((log(t)−β9)^2/2/β10^2)+β11/t/exp((log(t)−β12)^2/2/β13^2)) (2)

The variables *t*2 and *t*3 are time *t* to the second and third power; *β*_3_ and *β*_4_ are additional trend parameters.

For reweighting, binomial variances *var* = *fit*·(1−*fit*)/*LB* were used, where *fit* is the fitted value and *LB* is the number of live births. Two-sided statistical tests were applied, and p-values <0.05 were considered statistically significant. *R* statistical software version 4.2.2 was used for regression analyses and plotting graphs [15].

To determine the confidence limits for the estimated number of excess deaths in 1950–2000, the result for the regression parameter *β*_2_ in Model (1) was used. Because the research question is one-sided, i.e., whether there is increase-not decrease-in infant mortality, the 90% confidence interval (90% CI) for the number of excess deaths was determined. The 90% CI of the parameter *β*_2_ in Model (1) was calculated with the *R* function *confint*() which uses Monte Carlo simulation to estimate the upper and lower confidence limits. The results of regressions with parameter *β*_2_ fixed to the upper / lower limits were used to calculate the predicted infant deaths and the number of excess cases. The points in time of the maxima of the bell-shaped excess terms were calculated as exp (μ−*σ*^2^) where μ and σ are the parameters defining the lognormal function.

## Results

To start with, former studies of infant mortality in the U.S. shall be revisited. Sternglass compared the observed infant mortality rates after 1950 with the rates predicted from the extrapolated trend of the data in 1935–1950 to determine any excess infant mortality after the beginning of atmospheric nuclear weapon tests in Nevada in the early 1950s [5]. S5 Fig in S1 File shows the trend in infant mortality in the United States over the period 1934–2018 and the result of regressing the 1934–1950 data on an exponential model. The dashed line is the predicted trend for the period after 1950. For clarity, a semilogarithmic plot is used. Until the end of the study period, the observed infant mortality rates are well above the predicted trend. A purely exponential trend model is therefore inappropriate; it implies that infant mortality moves toward zero with time, which is not the case. As a result, Sternglass’ method overestimates the number of excess infant deaths.

Whyte determined the excess mortality in 1951–1980 by interpolation based on data before 1951 and after 1980 using an exponential trend model [12]. S6 Fig in S1 File shows the excess infant mortality rates obtained from Poisson regression of the 1934–1950 and 1981–1987 data with a linear time trend (panel A) and a linear-quadratic trend (panel B). The linear-quadratic trend model fits the data much better than the linear model. Thus, the exponential model used by Whyte is not compatible with the trend of the data before 1951 and after 1980.

Next, Poisson regression for the full study period (1934–2018) is carried out, again using a linear-quadratic time dependency for the undisturbed trend. The period 1950–2000 is chosen as the time window for estimating any excess mortality. From the difference between observed infant mortality rates and the rates predicted from regression without the 1950–2000 data, the number of excess infant deaths in 1950–2000 is estimated as 461,148. Regression with an additional cubic time variable does not notably improve the goodness of fit (p = 0.54). The regression result is shown in S7 Fig in S1 File.

### United States

**Fig 2:**
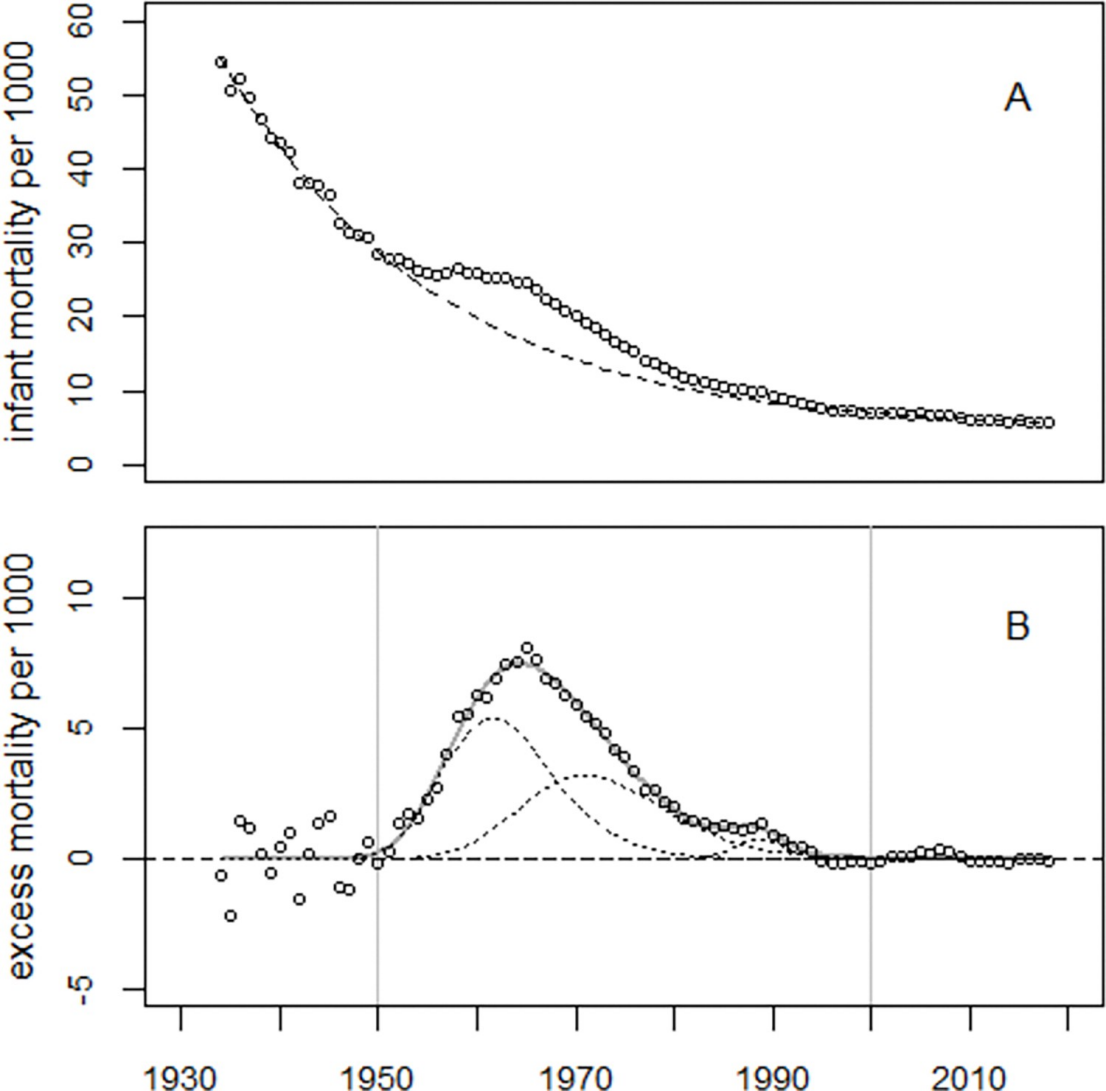
Panel A: Trend in infant mortality rates in the U.S. The dashed line shows the predicted undisturbed trend. Panel B: Excess infant mortality rates, i.e. observed- minus predicted rates and the result of regression with Model (1) (bold grey line). The thin dotted lines show the three bell-shaped excess terms. The vertical lines indicate the time window used to determine the number of excess infant deaths.

The data from the Unites States (U.S.) were first analyzed with Model (1). Regression without bell-shaped excess terms gave deviance = 43,881 (*df* = 82). With one excess term, the deviance was 3,244 (*df* = 79); a second excess term reduced the deviance to 2,363 (*df* = 76). A slight improvement in fit, although not statistically significant (p = 0.205), was achieved when the first excess term was replaced by a superposition of two excess terms with equal half-widths. The final Model (1) fitted the data well, albeit with large overdispersion; the deviance was 2,264 (*df* = 74), much greater than expected from a random distribution. From the difference between observed (O = 3,329,071) and predicted (E = 2,760,447) infant deaths during 1950–2000, the number of excess infant deaths was estimated as 568,624 (90% CI: 522,359 to 619,705). The O/E ratio was 1.206 (90% CI: 1.186 to 1.229). The trend of infant mortality rates and the excess rates are displayed in Fig 2.

**Fig 3:**
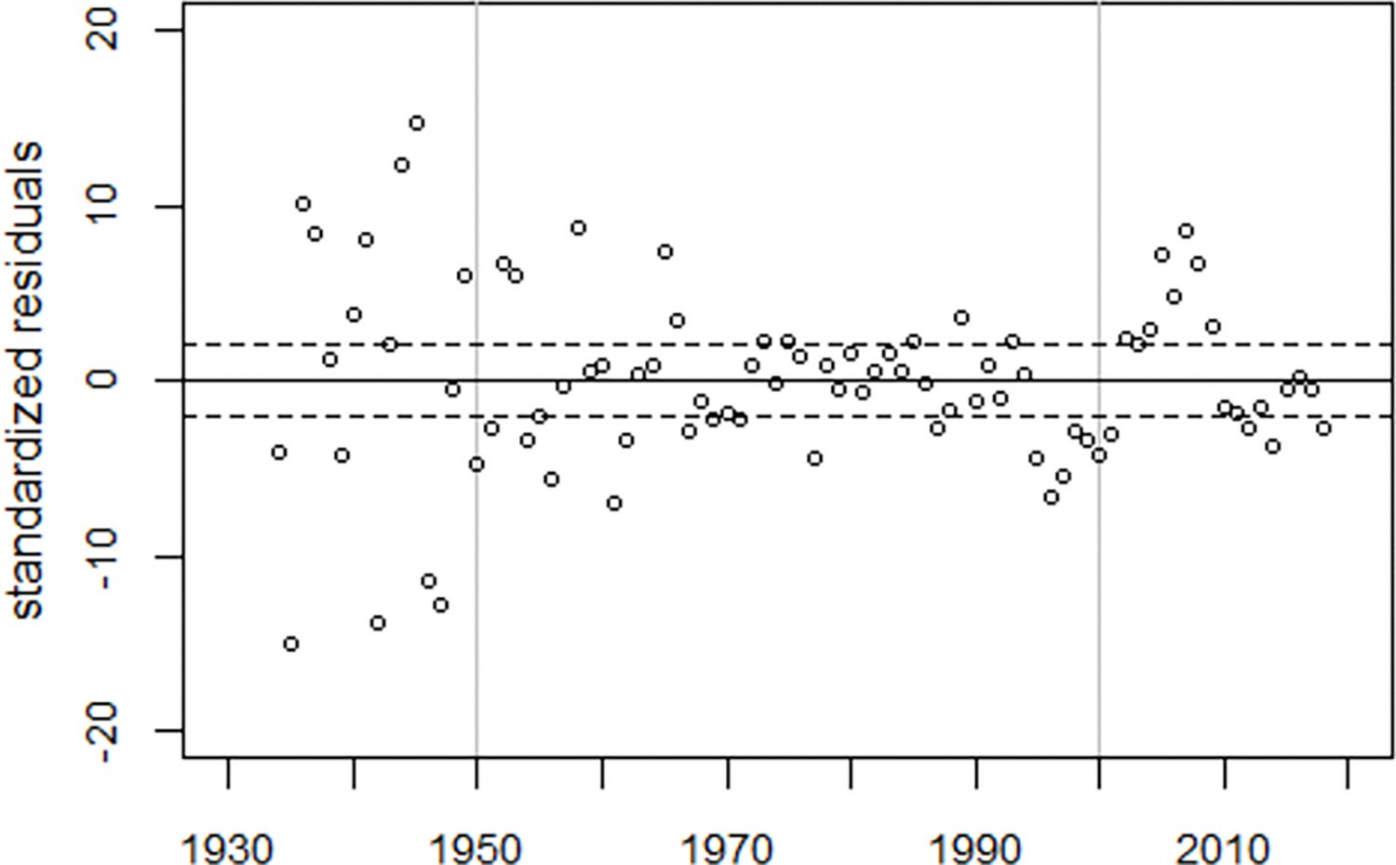
Deviations between observed and predicted infant mortality rates in units of standard deviations (standardized residuals) and range of ±2 standard deviations (broken lines).

To visualize the model fit, the deviations between the observed and fitted infant mortality rates were plotted in units of standard deviations (standardized residuals), see Fig 3. While there is no systematic deviation from the expected trend before 1995, the model does not fit the data thereafter, suggesting the existence of a fourth peak around 2007.

Model (2) was then applied to fit the U.S. data. Regression without excess terms gave deviance = 42,396 (*df* = 81); with one excess term the deviance was 2,998 (*df* = 78); the addition of a second excess term yielded deviance = 2,414 (*df* = 75), and the final Model (2) with 3 excess terms reduced the deviance to 2,352 (*df* = 73). 530,348 excess infant deaths were estimated during 1950–2000, an overall 19% increase. The maxima of the bell-shaped excess terms were found near 1960, 1970, and 1988. Table 1 contains the regression results (parameter estimates with standard errors and t-values) for the two regression models.

**Table 1:** Regression results for the United States with two competing trend models.

	Model (1)			Model (2)		
parameter	estimate	SE	t-value	estimate	SE	t-value
α	0.0048	0.0001	38.87			
β1	-2.717	0.014	-269.5	-2.729	0.013	-205.76
β2	-0.050	0.001	-44.20	-0.043	0.002	-27.84
β3				9.8E-05	4.2E-05	2.37
β4				9.0E-07	3.3E-07	2.70
β5	11.572	2.040	5.67	7.717	1.314	5.87
β6	3.534	0.048	73.55	3.508	0.041	84.89
β7	0.191	0.025	7.78	0.180	0.023	7.90
β8	14.492	2.956	4.90	9.349	1.929	4.85
β9	3.811	0.043	88.56	3.760	0.031	120.46
β10	(0.191)			(0.180)		
β11	10.202	2.283	4.47	4.673	1.129	4.14
β12	4.076	0.011	381.7	4.072	0.011	360.80
β13	0.045	0.013	3.49	0.043	0.013	3.24
	deviance = 2264 (*df* = 74)			deviance = 2352 (*df* = 73)		

Table 2 shows the improvement in fit resulting from stepwise increasing the number of excess terms for both regression models. Here, *OD* (overdispersion) is the deviance divided by the number of degrees of freedom (*df*2), and *df*1 is the number of parameters added with each step of model refinement.

**Table 2:** Improvement in fit to the U.S. data by stepwise model refinement.

Model (1)						
Excess terms	deviance	*df*2	OD^a^	*df*1	F-value^b^	p-value
0	43,881	82	535.1			
1	3,244	79	41.1	3	329.9	<0.001
2	2,363	76	31.1	3	9.44	<0.001
3	2,264	74	30.6	2	1.62	0.205
Model (2)						
0	42,396	81	523.4			
1	2,998	78	38.4	3	341.7	<0.001
2	2,414	75	32.2	3	6.04	0.001
3	2,352	73	32.2	2	0.96	0.388

^a^OD = deviance/*df*2

^b^F-value = (dev0-dev1)/*df*1/OD

### Europe

**Fig 4:**
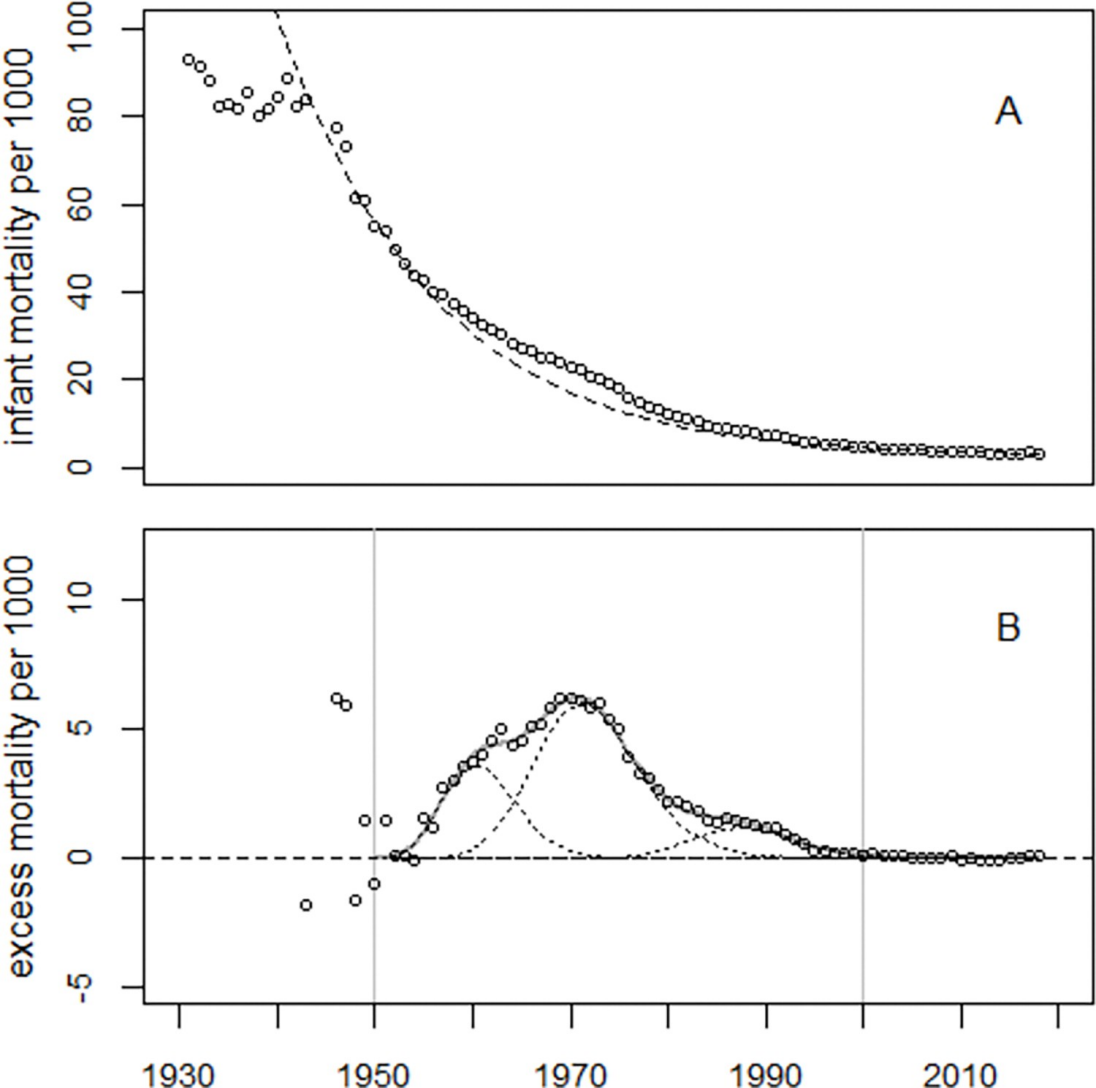
Panel A: Infant mortality rates in EU5 and predicted undisturbed trend (broken line). Panel B: Excess infant mortality rates, i.e. observed minus predicted rates and regression line.

The regression of infant mortality rates in Europe is complicated by the impact of the Second World War (WW2) on infant mortality. Here, the combined data from the United Kingdom (UK), Germany, France, Italy, and Spain, hereafter denoted as EU5, was analyzed from 1950 onward only, assuming that the effects of WW2 on infant mortality had been overcome by 1950. Regression with Model (1) without excess terms yielded deviance = 14,950 (*df* = 66); with one excess term, the deviance decreased to 2,245 (*df* = 63); a second excess term with the same width reduced the deviance to 1,642 (*df* = 61), and regression with the full Model (1) further reduced the deviance to 727 (df = 58). During 1950–2000, the estimated number of excess infant deaths was 559,370 (90% CI: 469,308 to 694,589), corresponding to an O/E ratio of 1.142 (90% CI: 1.117 to 1.183). Fig 4 shows the trend of infant mortality rates in EU5 (Panel A) and the excess infant mortality rates (Panel B).

**Fig 5:**
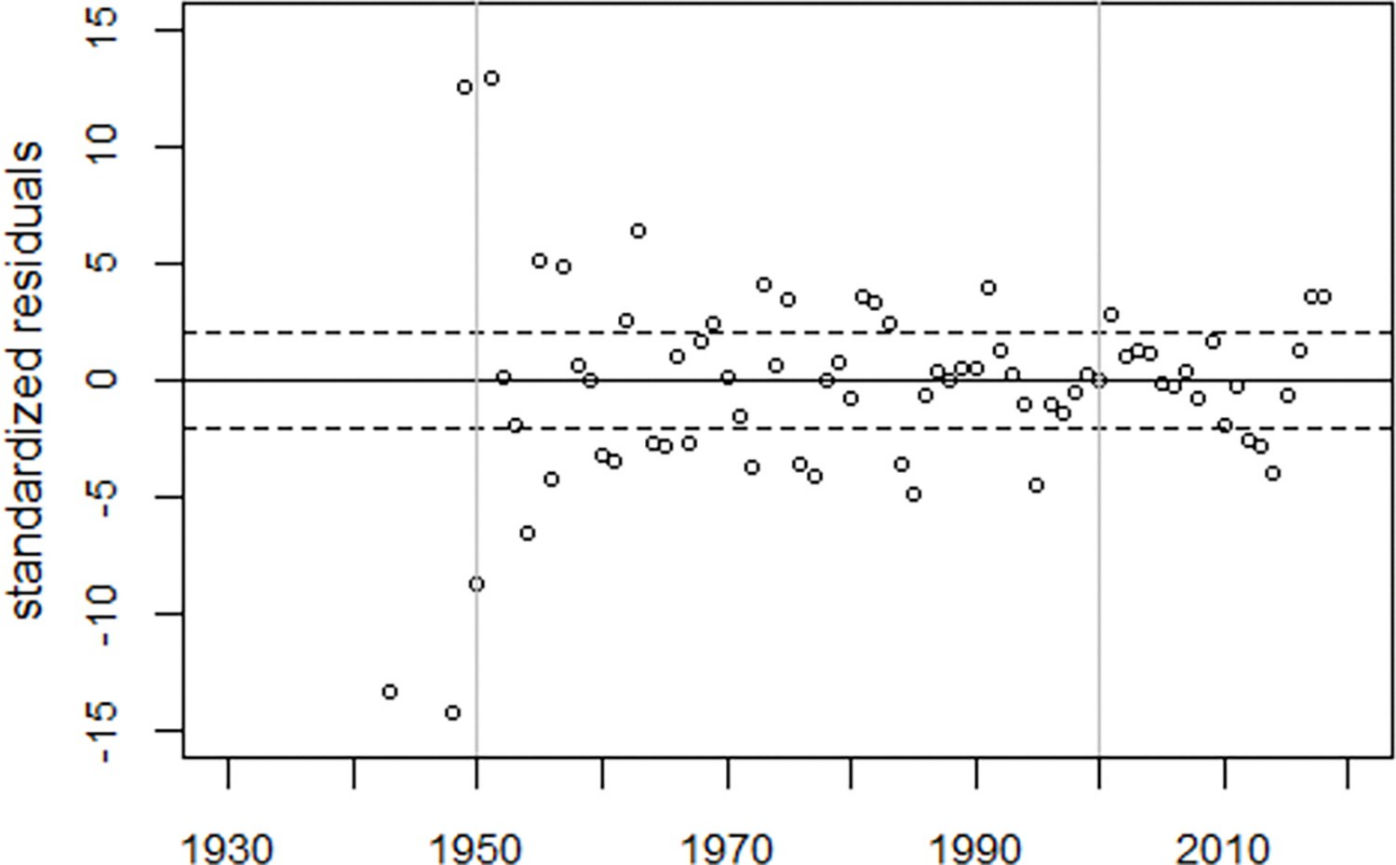
Deviations between observed and predicted infant mortality rates in units of standard deviations (standardized residuals) and range of ±2 standard deviations (broken lines).

Fig 5 shows the deviations between the observed and fitted infant mortality rates in units of standard deviations (standardized residuals). Over the period 1950–2018, the residuals exhibit no systematic deviations from the fitted model, i.e., the model fits the data well.

Regression with Model (2) without excess terms yielded deviance = 8,250 (*df* = 65). With three excess terms, the deviance decreased to 711 (*df* = 57). The number of excess infant deaths was estimated as 484,911, representing an overall increase in infant mortality of 12.1% during 1950–2000. The regression results for the two regression models are listed in Table 3.

**Table 3:** Regression results for the pooled five European countries (EU5), 1950–2018.

	Model (1)			Model (2)		
parameter	estimate	SE	t-value	estimate	SE	t-value
α	0.0027	0.0001	21.71			
β1	-1.510	0.045	-33.26	-1.714	0.137	-12.54
β2	-0.068	0.002	-30.32	-0.056	0.010	-5.60
β3				-2.2E-04	1.9E-04	-1.15
β4				3.8E-06	1.1E-06	3.43
β5	4.582	0.571	8.03	3.583	0.571	6.28
β6	3.473	0.009	370.68	3.470	0.012	281.72
β7	0.133	0.007	19.49	0.127	0.008	15.35
β8	17.538	1.843	9.52	12.830	1.270	10.10
β9	3.782	0.009	431.15	3.770	0.007	561.55
β10	(0.133)			(0.127)		
β11	15.667	3.619	4.33	7.672	1.204	6.37
β12	4.088	0.014	297.47	4.071	0.011	380.41
β13	0.099	0.015	6.71	0.087	0.013	6.74
	deviance = 727 (*df* = 58)			deviance = 711 (*df* = 57)		

Table 4 shows the improvement in fit with stepwise model refinement.

**Table 4:** Improvement in fit to the EU5 data by stepwise model refinement.

Model (1)						
excess terms	deviance	*df*2	OD^a^	*df*1	F-value^b^	p-value
0	14,950	66	226.5			
1	2,245	63	35.6	3	118.85	<0.001
2	1,642	61	26.9	2	11.21	<0.001
3	727	58	12.5	3	24.36	<0.001
Model (2)						
excess terms	deviance	*df*2	OD^a^	*df*1	F-value^b^	p-value
0	8,250	65	126.9			
1	1,836	62	29.6	3	72.19	<0.001
2	1,476	60	24.6	2	7.32	0.001
3	711	57	12.5	3	20.43	<0.001

^a^OD = deviance/*df*2

^b^F-value = (dev0-dev1)/*df*1/OD

Individual regressions were performed for the five European countries with Model (1). The parameter estimates with standard errors (SE) are listed in Tables 5 and 6. The numbers of observed (O) and predicted (E) infant deaths and respective excess deaths (O-E) during 1950–2000 are given in Table 7. The excess infant deaths add up to 547,352.

**Table 5:** Regression results for the three central European countries, 1950–2018.

	United Kingdom		Germany		France	
parameter	estimate	SE	estimate	SE	estimate	SE
α	0.0026	0.0002	0.0029	0.0001	0.0029	0.0001
β1	-2.590	0.023	-1.261	0.053	-1.457	0.056
β2	-0.046	0.001	-0.077	0.003	-0.074	0.002
β5	4.969	0.544	4.998	0.677	5.667	1.406
β6	3.534	0.014	3.439	0.005	3.611	0.024
β7	0.124	0.007	0.098	0.005	0.098	0.010
β8	16.551	1.093	20.390	2.880	10.910	2.067
β9	3.817	0.007	3.761	0.008	3.787	0.019
β10	(0.124)		(0.098)		(0.098)	
β11	11.147	1.643	25.120	4.299	28.190	4.599
β12	4.055	0.005	4.044	0.020	4.094	0.012
β13	-0.034	0.006	0.156	0.024	(0.098)	
deviance	258.7 (df = 58)		228.9 (df = 58)		585.8 (df = 59)	

**Table 6:** Regression results for the two southern European countries and EU5, 1950–2018.

	Italy		Spain		EU5	
parameter	estimate	SE	estimate	SE	estimate	SE
α	0.0019	0.0005	0.0015	0.0001	0.0027	0.0001
β1	-1.306	0.146	-1.346	0.024	-1.510	0.045
β2	-0.067	0.007	-0.063	0.001	-0.068	0.002
β5	6.158	1.650	3.875	0.631	4.582	0.571
β6	3.469	0.020	3.394	0.021	3.473	0.009
β7	0.133	0.016	0.121	0.013	0.133	0.007
β8	18.424	5.110	11.288	0.727	17.538	1.843
β9	3.759	0.024	3.649	0.014	3.782	0.009
β10	(0.133)		(0.121)		(0.133)	
β11	17.280	13.136	2.888	1.373	15.667	3.619
β12	4.103	0.046	3.865	0.027	4.088	0.014
β13	0.127	0.053	0.053	0.031	0.099	0.015
deviance	1052.0 (df = 58)		559.5 (df = 58)		726.5 (df = 58)	

**Table 7:** Observed (O) and predicted (E) infant deaths in five European countries, 1950–2000.

Country	O	E	O-E	O/E	deviance (df)
UK	663,769	593,243	70,526	1.119	258.7 (58)
Germany	1,153,522	1,007,707	145,815	1.145	228.9 (58)
France	750,766	692,148	58,618	1.085	585.8 (59)
Italy	1,124,657	943,153	181,504	1.192	1052.0 (58)
Spain	804,479	713,590	90,889	1.127	559.5 (58)
sum	4,497,193	3,949,841	547,352	1.139	

**Fig 6:**
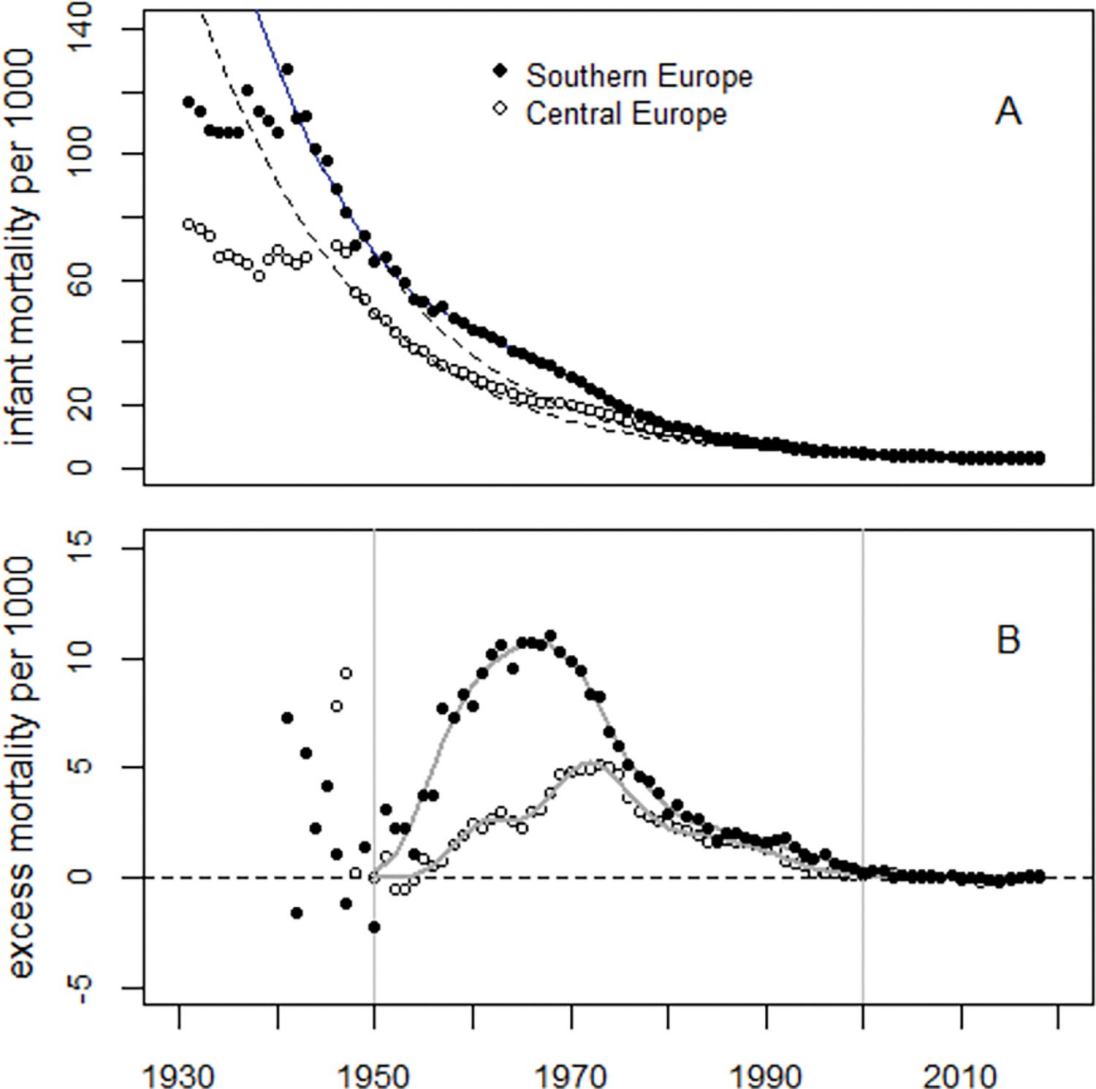
Panel A: Infant mortality rates in central- and southern Europe and predicted undisturbed trends (broken lines). Panel B: Excess infant mortality rates and regression lines.

To estimate any difference in effect size between central and southern European countries, infant mortality data from three central- (UK, Germany, and France) and two southern (Italy and Spain) European countries were analyzed separately with Model (1). The regression results are listed in Table 8. Interestingly, the estimates for the slope (parameter *β*_2_) were almost identical, and the estimates of the median values of the main two lognormal distributions agreed within the error bounds (see Table 8, parameters *β*_6_ and *β*_9_). In southern Europe, excess infant mortality rates were about twice as high as in central Europe (see Fig 6, Panel B).

**Table 8:** Results of regressions with Model (1) of the data from central- and southern Europe.

	Central Europe			Southern Europe		
parameter	estimate	SE	t-value	estimate	SE	t-value
α	0.0031	0.0001	27.78	0.0021	0.0004	5.86
β1	-1.637	0.051	-31.81	-1.233	0.136	-9.07
β2	-0.069	0.002	-28.30	-0.071	0.007	-9.94
β5	3.609	0.667	5.41	6.079	1.542	3.94
β6	3.479	0.012	293.4	3.462	0.020	169.1
β7	0.112	0.007	15.37	0.163	0.018	9.14
β8	18.310	1.877	9.75	18.262	5.635	3.24
β9	3.780	0.011	352.8	3.764	0.024	158.0
β10	(0.112)			(0.163)		
β11	20.793	4.048	5.14	19.349	13.857	1.40
β12	4.069	0.013	302.1	4.132	0.040	102.5
β13	0.108	0.016	6.97	0.125	0.040	3.11
	deviance = 511 (*df* = 58)			deviance = 812 (*df* = 58)		

The estimated numbers of excess infant deaths during 1950–2000 were 269,371 (90% CI: 77,392 to 337,039) in central Europe and 356,878 (90% CI: 256,526 to 667,625) in southern Europe, totaling 626,249 in EU5.

### Sensitivity analyses

#### United Kingdom

The data from the United Kingdom show little overdispersion and lend themselves to more detailed exploratory analysis. Model (1) is supplemented by two additional bell-shaped excess terms. The fourth term reduces the deviance from 259 (df = 58) to 201.6 (df = 55), p = 0.003, and a fifth excess term yields deviance = 143.5 (df = 52), p = 0.0005. Table 9 shows the improvement in fit by gradually increasing the number of excess terms.

**Table 9:** Improvement in fit to the UK data by stepwise model refinement.

Excess terms	deviance	*df*2	OD^a^	*df*1	F-value^b^	p-value
0	3,673	66	55.7			
1	556.5	63	8.8	3	117.60	<0.0001
2	500.1	61	8.2	2	3.44	0.0385
3	258.7	58	4.5	3	18.05	<0.0001
4	201.6	55	3.7	3	5.19	0.0031
5	143.5	52	2.8	3	7.01	0.0005

^a^OD = deviance/*df*2

^b^F-value = (dev0-dev1)/*df*1/OD

**Fig 7:**
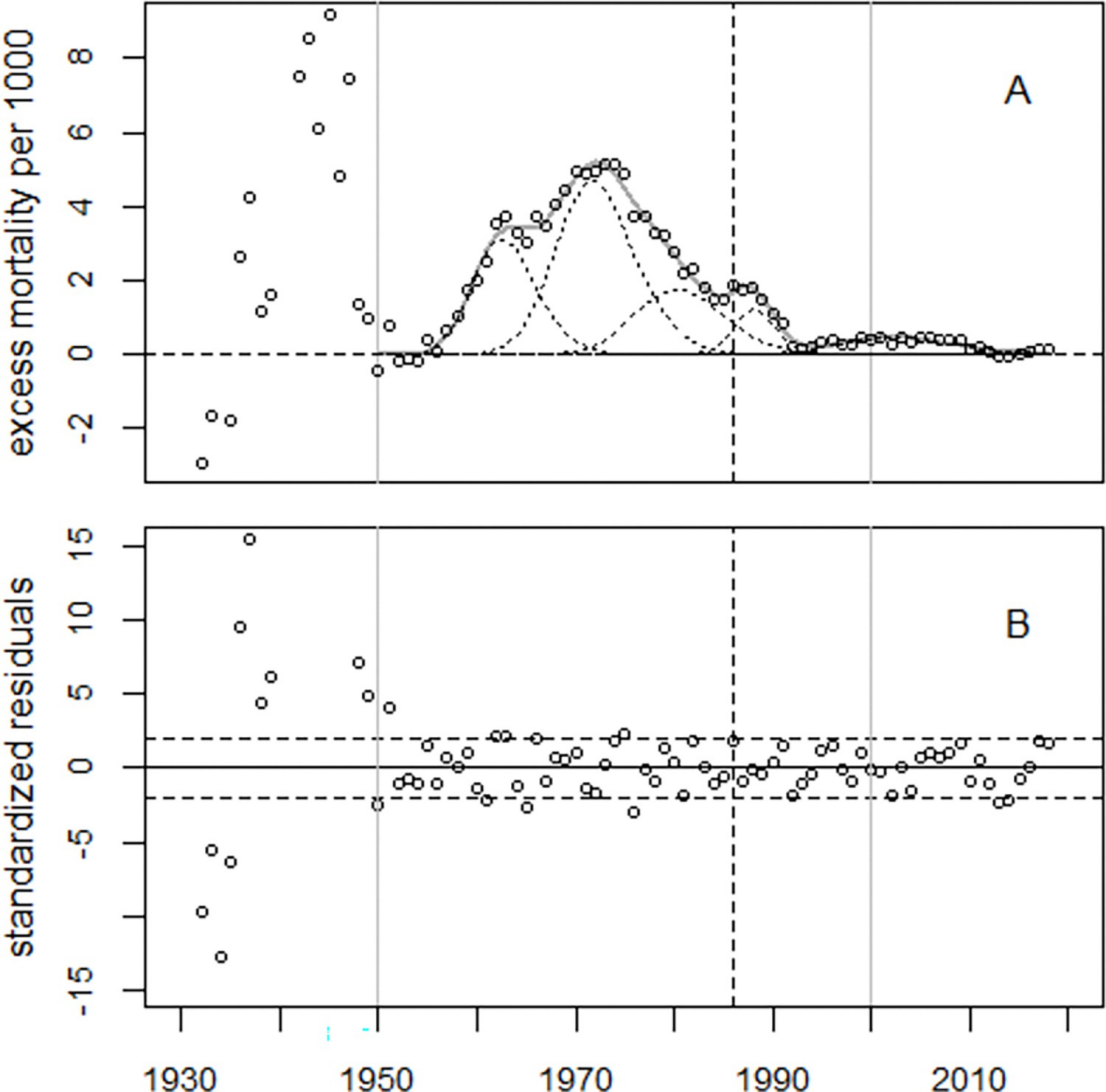
Panel A: Excess infant mortality rates in the UK and regression line. The thin dotted lines show the five excess terms. Panel B: Standardized residuals. The broken vertical line indicates 1986, the year of the Chernobyl accident.

Fig 7 panel A shows the trend of the excess infant mortality rates in the UK, and the thin dotted lines show the five excess terms. Panel B displays the residuals in units of standard deviations. The narrow peak around 1988 could be a consequence of the Chernobyl accident. The number of excess infant deaths in 1986–1992 is estimated as 4,500 with a maximum number of 1,000 excess cases in 1988.

The estimated number of excess infant deaths during 1950–2000 depended on the model; for three excess terms, it was 70,527 (64,392 to 77,393), and for five excess terms, the number increased to 86,990 (73,345 to 110,433).

**Sweden.** The Swedish data are interesting for two reasons: (a) Sweden was not involved in the Second World War; (b) the intensity of strontium-90 fallout from nuclear weapons tests depended on the latitude [16]; in the northern hemisphere, it peaked between latitudes 40°N and 50°N, see S8 Fig in S1 File. Stockholm is situated at latitude 60°N, London 51.5°N, Berlin 52°N, Munich 48°N, Paris 49°N, Rome 42°N, and Madrid 40°N. If the excess infant mortality depends on the strontium deposition, the effect in Sweden should be smaller than that in EU5.

The Swedish data was analyzed with the same regression model used above for the UK data, i.e. with Model (1) and five excess terms. The regression results are presented in S1 Table in S1 File, together with the results for the United Kingdom. S9 Fig in S1 File shows infant mortality rates in Sweden together with those from EU5. The mean excess rate in Sweden is smaller than that in EU5 which is consistent with the latitudinal dependence of strontium-90 deposition from global fallout. As in the data from the UK, there is a peak after the Chernobyl accident which may be related to the consumption of contaminated meat from reindeer. The number of excess infant deaths attributable to this spike is 276 in 1986–1994, with a maximum of 127 excess cases in 1991 alone.

### Analysis of European data, 1931–2018

To avoid trend bias due to the impact of WW2, the above analyses of European infant mortality data were restricted to data after 1949. As an alternative, the rise in infant mortality during and after WW2 is approximated by an additional bell-shaped excess term. This new model, i.e. Model (2), supplemented by an additional bell-shaped term, will hereafter be referred to as Model (3).

**Fig 8:**
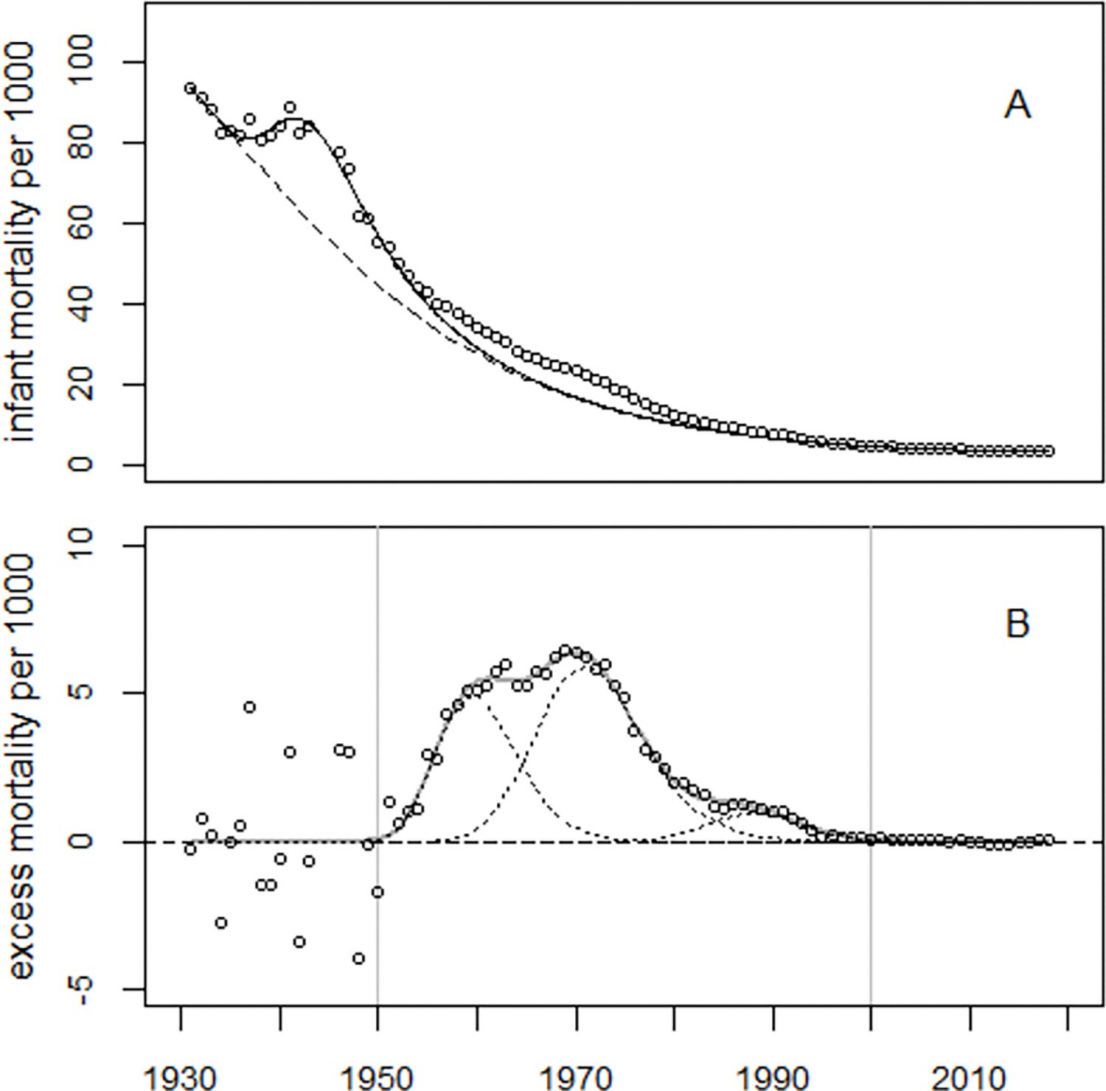
Panel A: Infant mortality rates in EU5, 1931–2018. The interrupted line shows the unperturbed secular trend; the solid line shows the predicted trend adjusted for the effects of WW2. Panel B: Excess infant mortality rates and regression line.

**Fig 9:**
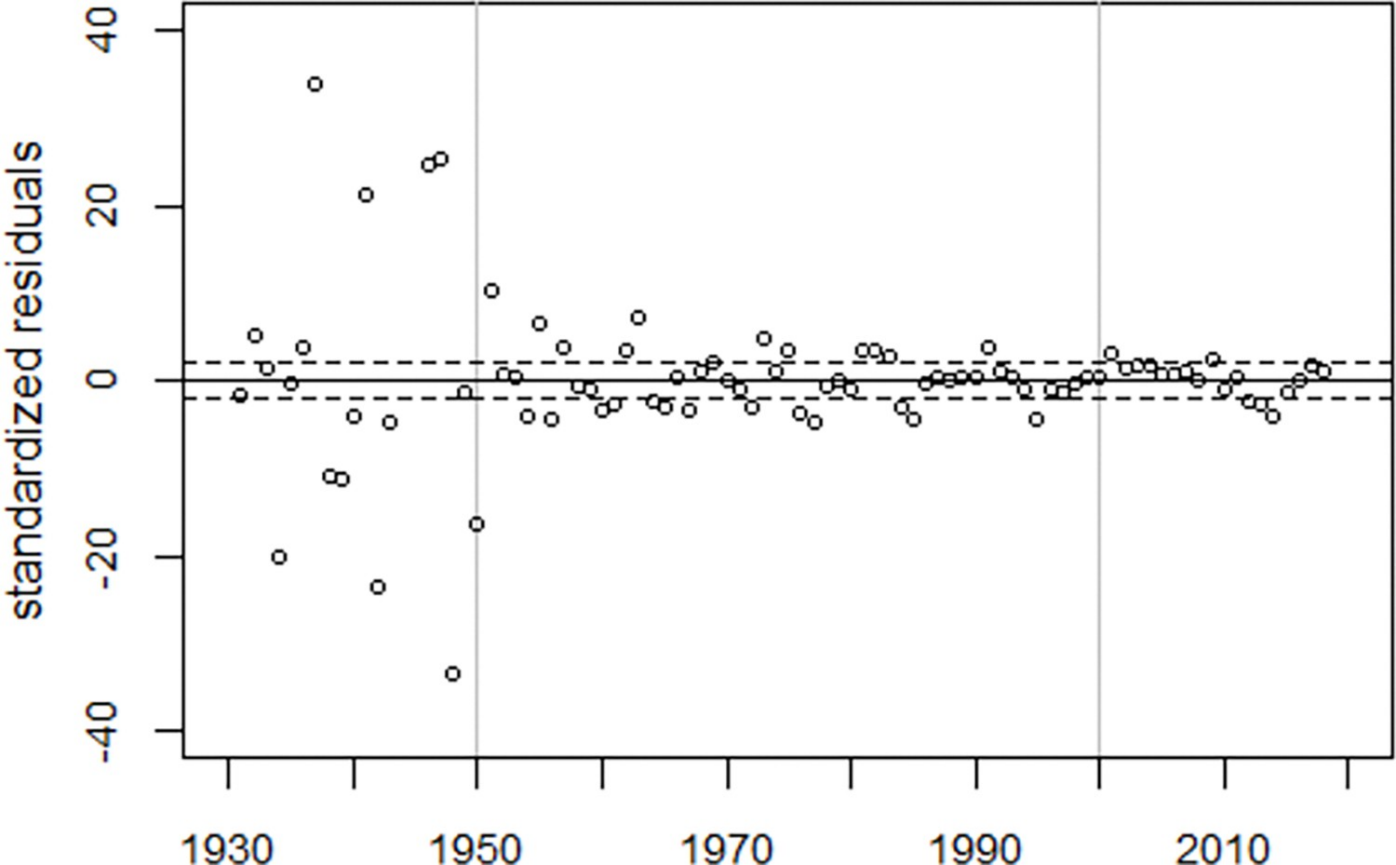
Observed minus fitted infant mortality rates in EU5 in units of standard deviations (standardized residuals) and the range of ±2 standard deviations (broken lines).

Model (3) fitted the EU5 data well albeit with large overdispersion (deviance = 6,120, *df* = 71), see Figs 8 and 9. The regression results for EU5 are listed in S2 Table in S1 File. The number of excess infant deaths during 1950–2000 was estimated as 617,621. A likewise extended Model (1) fitted the data less well; the deviance was 6,714 (*df* = 72), much greater than the deviance for Model (3). The number of excess infant deaths in 1950–2000 increased to 819,573, exceeding by far the respective figure obtained with Model (2).

Regressions with Model (3) were also conducted for each of the five European countries, see S10-14 Figs in S1 File. To analyze the German data, a second bell-shaped excess term was needed to fit the data before 1950. In France, highly significant peaks in infant mortality were found in 1940 and 1945; these two years were therefore omitted from the regression. The individual regression results for the five European countries are listed in S3, S4 Tables in S1 File. Table 10 shows the number of excess infant deaths in individual countries.

**Table 10:** Observed and predicted infant deaths in 1950–2000 from regressions with Model (3).

Country	O	E	O-E	O/E	Deviance (df)
UK	663,769	579,816	83,953	1.145	2,422 (73)
Germany	1,153,522	1,018,507	135,015	1.133	2,787 (69)
France	750,766	679,402	71,364	1.105	1,688 (71)
Italy	1,124,657	979,692	144,965	1.148	4,833 (73)
Spain	804,479	698,944	105,535	1.151	7,334 (73)
sum	4,497,193	3,956,361	540,832	1.137	

Regression of the Swedish data on infant mortality, 1931–2018, was conducted with Model (3) as well. Highly significant drops in infant mortality were detected in 1942 and 1943 with deviations of more than four standard deviations from the predicted trend; infant mortality rates in these two years were considered outliers and omitted from the regression. The estimated number of excess infant deaths in 1950–2000 was 9,598, an overall 20% increase. The trend of the infant mortality rates from Sweden, the excess rates, and the residuals are shown in S15 Fig in S1 File.

### Effect of gender

To check whether the excess infant mortality depends on gender, the data from the U.S. (1936–2018) and from the UK (1950–2018) were analyzed for male and female infants separately with Model (1) and three bell-shaped excess terms. For males and females, the points in time of the maxima (median values) as well as the widths (geometric standard deviations) and the relative excess mortalities agreed within the limits of error for the two main excess terms with peaks around 1960 and 1970. However, the peak in the late 1980s was greater for males than for females. The results for the United States and the United Kingdom are listed in S5 and S6 Tables in S1 File. The respective graphs are shown in S16, S17 Figs in S1 File.

### Trend analysis of German stillbirth rates

**Fig 10:**
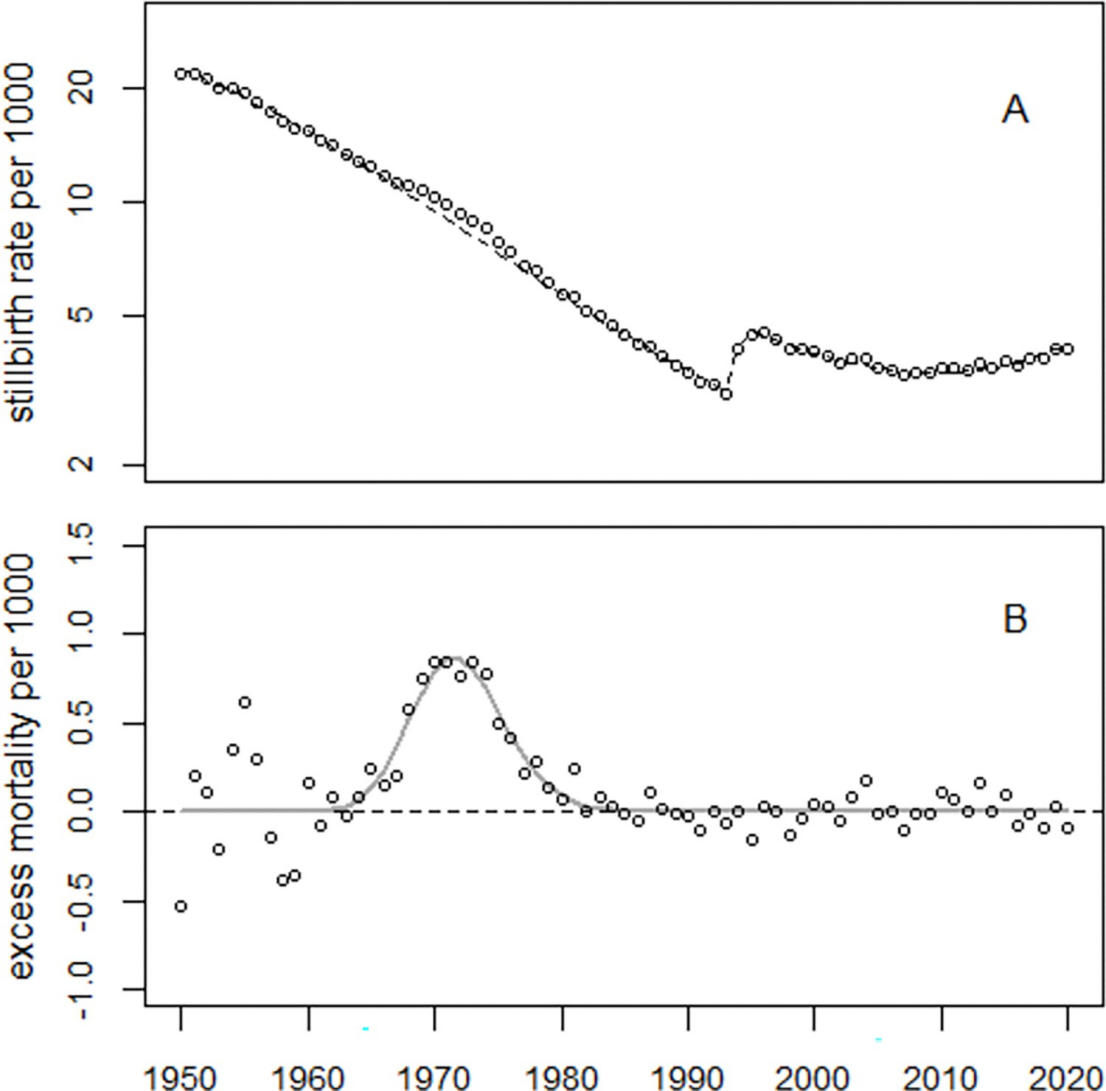
Panel A: Stillbirth rates in Germany, semilogarithmic plot, and reduced model (broken line). Panel B: Excess stillbirth rates and regression line.

**Fig 11:**
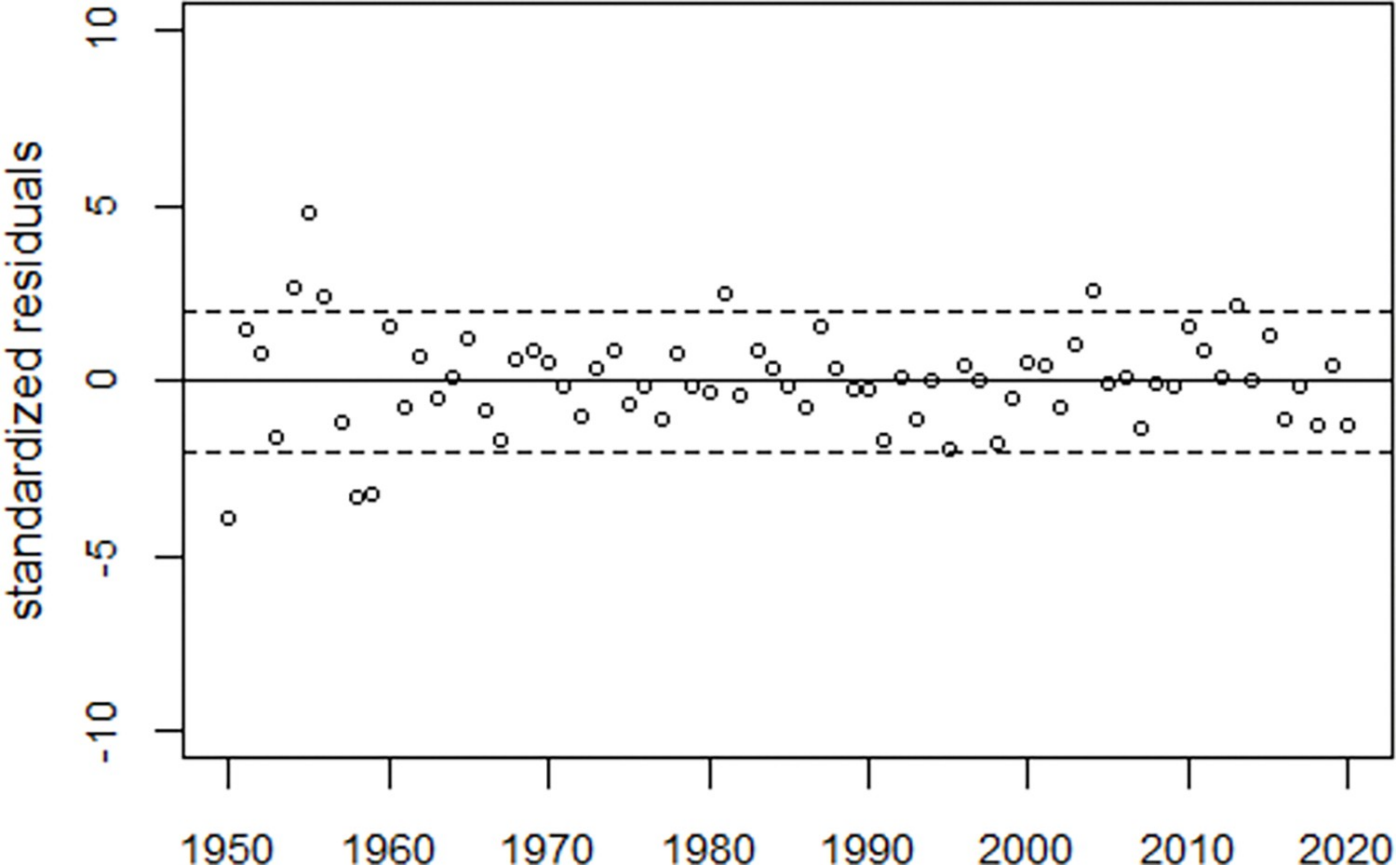
Standardized residuals from regression of stillbirth rates in Germany and range of ±2 standard deviations (broken lines).

Since annual data of livebirths and stillbirths from Germany are also provided in [14], a trend analysis of stillbirth rates in the period 1950–2020 was performed using regression Model (2). In 1994, the definition of stillbirth was changed from a birth weight of >1000 grams to >500 grams, so stillbirth rates after 1993 were adjusted by adding two dummy variables that allowed for an increase in 1994 and a level shift in the period 1995–2020. The regression model used a third-degree polynomial for the time dependence and a single bell-shaped excess term. The model fitted the data well, see Figs 10 and 11; the deviance was 140.4 (df = 62), much less than deviance = 417.8 (df = 65) obtained from regression without the excess term. The median of the lognormal function was 1972.5 (95% CI: 1971.7 to 1973.3). The deviation of stillbirth rates from the predicted trend corresponds to 7,785 excess stillbirths.

### Infant mortality in Russia

Out of curiosity, data on infant mortality in Russia was also examined. On the mortality.org website, data on live births and infant deaths from Russia are only available from 1959 onwards, so that a meaningful study of the possible effects of nuclear weapons testing is not possible. However, infant mortality rates show a relative maximum in 1976, with a decreasing trend thereafter. An analysis of infant mortality rates from 1976 to 2010 exhibits an upward shift in 1984 and a long-term increase and decrease after the Chernobyl accident in 1986 relative to rates predicted from the pre-Chernobyl trend. The deviation of observed rates from the predicted trend can be approximated by two lognormal distributions with maxima around 1994 and 2001. The excess of infant mortality rates corresponds to 87,436 additional infant deaths during 1987–2010 (see S18 Fig in S1 File).

### Discussion

The present study examines the trends in infant mortality in the United States (U.S.), and five major European countries combined (EU5) before and after atmospheric nuclear weapons testing. in the 1950s and early 1960s. In both regions, deviations from a uniformly decreasing trend are found in the period 1950–2000. These deviations were approximated by three superimposed bell-shaped excess terms (lognormal distributions). The median values of the two main lognormal distributions agree within the error limits for the U.S. and the EU5 (see parameters *β*_6_, *β*_9_ in Tables 1 and 3). This also holds for the comparison of the results for central Europe (UK, Germany, France) and southern Europe (Italy and Spain), see Table 8.

The point in time of the peak in excess infant mortality depends on the relative size of the two main excess terms with maxima in the early1960s and 1970s. In the U.S., the first excess term is greater than the second, so the overall maximum appears earlier than in EU5 where the second excess term dominates (cf. Figs 2 and 4).

From the regression results for individual European countries in Tables 5 and 6, the timing of the maxima for the first and second lognormal distributions was calculated, see Table 11. In the United Kingdom, both peaks are lagged compared to the corresponding peaks in EU5, while in Spain, both peaks occur earlier than in EU5. Differences in the age distribution of mothers in the United Kingdom and Spain could explain this observation; Spanish women in the 1960s and 1970s may have been younger at their first pregnancy than women from the United Kingdom, but this could not be verified because the author did not have historical data on the age distribution of mothers.

**Table 11:** Estimated timing (calendar year) of the two main infant mortality peaks.

	First peak	Second peak
Country	Peak position (95% CI)	Peak position (95% CI)
UK	1963.7 (1962.8, 1964.7)	1974.8 (1974.1, 1975.4)
Germany	1960.9 (1960.5, 1961.2)	1972.6 (1971.9, 1973.3)
France	1966.7 (1964.9, 1968.5)	1973.7 (1972.1, 1975.4)
Italy	1961.5 (1960.3, 1962.8)	1972.2 (1970.2, 1974.2)
Spain	1959.4 (1958.1, 1960.6)	1967.9 (1966.9, 1968.9)
EU5	1961.7 (1961.1, 1962.3)	1973.1 (1972.4, 1973.9)

The two main peaks in infant mortality follow, with a lag of about seven years, the fallout peaks after the two largest atmospheric weapons tests: the U.S. hydrogen bomb "Castle Bravo" at Bikini Atoll, March 1, 1954, with an explosive force equivalent to 15 million tons (Mt) of TNT, and the largest Soviet hydrogen bomb "Tsar Bomba" at Novaya Zemlya, October 30, 1961, with 50 Mt TNT. The dominance of the first peak in the U.S. may be explained by the Nevada tests and higher exposure to fallout from the Pacific tests in the 1950s.

Both Sternglass and Whyte had suggested strontium in the fallout as a likely cause of the observed increased infant mortality [5, 12, 17]. However, no direct relationship between annual strontium exposure and increased infant mortality has been demonstrated [11]. Körblein proposed a possible mechanism to explain the observed long-term deviations of perinatal mortality from a uniform declining trend [13, 18]. This mechanism, a delayed effect of strontium on the immune system, was used to model the trend of infant mortality rates in the present study.

The main limitation of the study is that we do not know how infant mortality trends would have evolved in the absence of the effects of atmospheric weapons testing. Improvements in health care, such as the introduction of prenatal ultrasonography in the early 1980s, and socioeconomic influences likely affect infant mortality trends. The results must therefore be interpreted with due caution. However, the rates of stillbirths in Germany (1950–2020) follow a remarkably smooth trend, with the exception of a bell-shaped increase between 1960 and 1980 (see Fig 10).

## Conclusion

The present analysis of infant mortality after atmospheric nuclear weapons testing shows similar trends in the United States and Europe. The same statistical model was used to fit the data in both regions: a uniformly decreasing trend with three superimposed bell-shaped excess terms. The increased infant mortality is interpreted as a delayed effect of strontium on the immune system. However, this hypothesis cannot be tested because there are no data on strontium exposure in pregnant women. Acceptance of the strontium hypothesis means that atmospheric weapons testing could be responsible for the deaths of millions of infants in the Northern Hemisphere.

## Supporting information

S1 File (DOCX)

S1 Data (XLSX)
