# Supplementary material for: Statistical modeling of trends in infant mortality after atmospheric nuclear weapons testing
Source: PLoS One. 2023 May 18;18(5):e0284482. doi: 10.1371/journal.pone.0284482 (PMC10194983; doi:10.1371/journal.pone.0284482)

**Supporting Information**

(Statistical modeling of trends in infant mortality after atmospheric nuclear weapons testing)

S1 Table. Results for U.K. and Sweden 1950-2018, 5 excess terms

|  | United Kingdom | | | Sweden | | |
| --- | --- | --- | --- | --- | --- | --- |
| parameter | estimate | SE | t-value | estimate | SE | t-value |
| α | 0.0027 | 0.0002 | 14.12 | 0.0018 | 0.0003 | 5.65 |
| β1 | -2.507 | 0.058 | -43.23 | -2.744 | 0.172 | -15.96 |
| β2 | -0.050 | 0.003 | -17.48 | -0.057 | 0.009 | -6.44 |
| β5 | 6.219 | 0.798 | 7.80 | 8.010 | 2.167 | 3.70 |
| β6 | 3.507 | 0.013 | 280.52 | 3.467 | 0.019 | 177.95 |
| β7 | 0.101 | 0.011 | 9.31 | 0.124 | 0.018 | 6.95 |
| β8 | 16.973 | 5.354 | 3.17 | 15.750 | 5.757 | 2.74 |
| β9 | 3.759 | 0.040 | 94.80 | 3.746 | 0.033 | 114.00 |
| β10 | (0.101) |  |  | (0.124) |  |  |
| β11 | 12.713 | 4.471 | 2.84 | 32.432 | 16.411 | 1.98 |
| β12 | 3.944 | 0.034 | 115.23 | 4.086 | 0.027 | 150.21 |
| β13 | 0.093 | 0.048 | 1.95 | 0.124 | 0.030 | 4.15 |
| β14 | 14.163 | 5.219 | 2.71 | 15.784 | 4.188 | 3.77 |
| β15 | 4.066 | 0.005 | 841.56 | 4.112 | 0.005 | 837.01 |
| β16 | 0.036 | 0.009 | 4.09 | 0.017 | 0.006 | 2.90 |
| β17 | 14.778 | 6.687 | 2.21 | 21.189 | 11.173 | 1.90 |
| β18 | 4.308 | 0.020 | 217.56 | 4.270 | 0.009 | 487.24 |
| β19 | 0.080 | 0.033 | 2.44 | 0.018 | 0.010 | 1.75 |
|  | deviance=143.5 (df=52) | | | deviance=73.1 (df=52) | | |

S2 Table. Regression results for Europe (EU5), 1931-2018

|  | Modified Model (1) | | | Model (3) | | |
| --- | --- | --- | --- | --- | --- | --- |
| parameter | estimate | SE | t-value | estimate | SE | t-value |
| α | 0.0023 | 0.0004 | 6.155 |  |  |  |
| β1 | -2.215 | 0.010 | -230.164 | -2.341 | 0.016 | -143.188 |
| β2 | -0.055 | 0.004 | -14.867 | -0.029 | 0.006 | -4.959 |
| β3 |  |  |  | -6.1E-04 | 1.5E-04 | -4.053 |
| β4 |  |  |  | 5.7E-06 | 1.1E-06 | 5.171 |
| β5 | 7.522 | 1.400 | 5.374 | 4.872 | 1.095 | 4.447 |
| β6 | 3.455 | 0.019 | 180.953 | 3.440 | 0.020 | 173.691 |
| β7 | 0.149 | 0.014 | 10.784 | 0.140 | 0.013 | 10.458 |
| β8 | 22.045 | 4.718 | 4.672 | 13.980 | 2.538 | 5.507 |
| β9 | 3.785 | 0.020 | 185.305 | 3.768 | 0.016 | 236.064 |
| β10 | (0.149) |  |  | (0.140) |  |  |
| β11 | 14.026 | 7.274 | 1.928 | 7.558 | 2.943 | 2.568 |
| β12 | 4.082 | 0.029 | 139.202 | 4.079 | 0.026 | 156.804 |
| β13 | 0.088 | 0.033 | 2.641 | 0.081 | 0.032 | 2.577 |
| β14 | 9.468 | 1.228 | 7.713 | 5.440 | 0.836 | 6.509 |
| β15 | 2.969 | 0.058 | 51.225 | 2.821 | 0.048 | 58.904 |
| β16 | 0.487 | 0.030 | 16.088 | 0.382 | 0.035 | 10.862 |
|  | deviance=6714 (df=72) | | | deviance=6120 (df=71) | | |

S3 Table. Results for the three central European countries, 1931-2018

|  | U.K. | | Germany | | France | |
| --- | --- | --- | --- | --- | --- | --- |
| parameter | estimate | SE | estimate | SE | estimate | SE |
| β1 | -2.588 | 0.017 | -2.441 | 0.027 | -2.484 | 0.017 |
| β2 | -0.049 | 0.005 | -0.058 | 0.011 | -0.019 | 0.005 |
| β3 | 1.2E-04 | 1.5E-04 | 1.3E-04 | 2.4E-04 | -9.2E-04 | 1.3E-04 |
| β4 | 4.9E-07 | 1.1E-06 | 1.2E-06 | 1.5E-06 | 8.3E-06 | 1.0E-06 |
| β5 | 5.311 | 1.287 | 3.977 | 1.582 | 2.333 | 0.908 |
| β6 | 3.511 | 0.034 | 3.448 | 0.018 | 3.216 | 0.053 |
| β7 | 0.130 | 0.018 | 0.099 | 0.010 | 0.151 | 0.026 |
| β8 | 14.720 | 1.922 | 19.500 | 2.635 | 6.974 | 2.400 |
| β9 | 3.809 | 0.017 | 3.770 | 0.011 | 3.714 | 0.027 |
| β10 | (0.130) |  | (0.099) |  | (0.151) |  |
| β11 | 7.915 | 2.852 | 12.420 | 3.065 | 13.180 | 3.095 |
| β12 | 4.055 | 0.014 | 4.008 | 0.030 | 4.093 | 0.014 |
| β13 | 0.034 | 0.016 | (0.099) |  | 0.073 | 0.018 |
| β14 | 3.367 | 0.533 | 6.759 | 0.521 | 5.843 | 0.495 |
| β15 | 2.611 | 0.059 | 2.774 | 0.008 | 2.746 | 0.022 |
| β16 | 0.344 | 0.050 | 0.099 | 0.010 | 0.244 | 0.023 |
| β17 |  |  | 16.660 | 4.304 |  |  |
| β18 |  |  | 3.343 | 0.157 |  |  |
| β19 |  |  | 0.556 | 0.075 |  |  |
| deviance | 2,422 (df=73) | | 2,787 (df=69) | | 1,688 (df=71) | |

S4 Table. Results for the two southern European countries and EU5, 1931-2018

|  | Italy | | Spain | | EU5 | |
| --- | --- | --- | --- | --- | --- | --- |
| parameter | estimate | SE | estimate | SE | estimate | SE |
| β1 | -2.230 | 0.021 | -2.082 | 0.033 | -2.341 | 0.016 |
| β2 | 0.001 | 0.006 | -0.017 | 0.014 | -0.029 | 0.006 |
| β3 | -1.5E-03 | 2.9E-04 | -9.5E-04 | 3.8E-04 | -6.1E-04 | 1.5E-04 |
| β4 | 1.2E-05 | 2.5E-06 | 7.4E-06 | 2.7E-06 | 5.7E-06 | 1.1E-06 |
| β5 | 4.174 | 1.505 | 1.918 | 2.062 | 4.872 | 1.095 |
| β6 | 3.491 | 0.041 | 3.261 | 0.176 | 3.440 | 0.020 |
| β7 | 0.113 | 0.025 | 0.172 | 0.063 | 0.140 | 0.013 |
| β8 | 13.260 | 3.731 | 9.999 | 4.432 | 13.980 | 2.538 |
| β9 | 3.743 | 0.042 | 3.641 | 0.043 | 3.768 | 0.016 |
| β10 | (0.113) |  | (0.172) |  | (0.140) |  |
| β11 | 17.140 | 10.320 | 5.127 | 7.624 | 7.558 | 2.944 |
| β12 | 4.118 | 0.060 | 4.121 | 0.063 | 4.079 | 0.026 |
| β13 | 0.167 | 0.092 | 0.038 | 0.073 | 0.081 | 0.032 |
| β14 | 3.599 | 0.283 | 3.285 | 0.971 | 5.440 | 0.836 |
| β15 | 2.590 | 0.013 | 2.388 | 0.099 | 2.821 | 0.048 |
| β16 | 0.177 | 0.017 | 0.344 | 0.077 | 0.382 | 0.035 |
| deviance | 4,833 (df=73) | | 7,334 (df=73) | | 6,120 (df=71) | |

S5 Table. Results for infant mortality in the United States by gender

|  | males | | | females | | |
| --- | --- | --- | --- | --- | --- | --- |
| parameter | estimate | SE | t-value | estimate | SE | t-value |
| α | 0.0054 | 0.0001 | 38.18 | 0.0047 | 0.0001 | 38.18 |
| β1 | -2.610 | 0.013 | -202.78 | -2.840 | 0.016 | -202.77 |
| β2 | -0.049 | 0.001 | -37.95 | -0.052 | 0.002 | -37.95 |
| β5 | 12.229 | 3.532 | 3.46 | 14.774 | 3.821 | 3.46 |
| β6 | 3.553 | 0.076 | 46.86 | 3.576 | 0.067 | 46.86 |
| β7 | 0.208 | 0.033 | 6.36 | 0.215 | 0.030 | 6.36 |
| β8 | 14.304 | 4.685 | 3.05 | 15.595 | 4.154 | 3.05 |
| β9 | 3.819 | 0.059 | 64.59 | 3.885 | 0.083 | 64.59 |
| β10 | (0.208) |  |  | (0.215) |  |  |
| β11 | 11.289 | 2.174 | 5.19 | 10.634 | 3.026 | 5.19 |
| β12 | 4.077 | 0.009 | 464.98 | 4.081 | 0.012 | 464.98 |
| β13 | 0.046 | 0.009 | 5.02 | (0.046) |  |  |
| parameter | estimate | SE | t-value | difference | SE | t-value |
| α | 0.0054 | 0.0001 | 38.18 | -0.0007 | 0.0002 | -3.76 |
| β1 | -2.610 | 0.013 | -202.78 | -0.231 | 0.021 | -11.11 |
| β2 | -0.049 | 0.001 | -37.95 | -0.003 | 0.002 | -1.32 |
| β5 | 12.229 | 3.532 | 3.46 | 2.545 | 4.781 | 0.53 |
| β6 | 3.553 | 0.076 | 46.86 | 0.022 | 0.094 | 0.24 |
| β7 | 0.208 | 0.033 | 6.36 | 0.007 | 0.043 | 0.15 |
| β8 | 14.304 | 4.685 | 3.05 | 1.291 | 5.800 | 0.22 |
| β9 | 3.819 | 0.059 | 64.59 | 0.065 | 0.093 | 0.70 |
| β10 | (0.208) |  |  | (0.007) |  |  |
| β11 | 11.289 | 2.174 | 5.19 | -0.655 | 3.700 | -0.18 |
| β12 | 4.077 | 0.009 | 464.98 | 0.005 | 0.014 | 0.32 |
| β13 | 0.046 | 0.009 | 5.02 | (0) |  |  |

Deviance= 1990.4 (df=145), ^1^pvalue for difference between estimates for males and females

To determine whether the estimates of the parameters for females differ from those for males, parameters β in the regression model are replaced by β+δ (beta plus delta). Parameter δ is then tested for statistical significance with H0: δ=0.

S6 Table. Results for infant mortality in the United Kingdom by gender

|  | males | | | females | | |  |
| --- | --- | --- | --- | --- | --- | --- | --- |
| parameter | estimate | SE | t-value | estimate | SE | t-value |  |
| α | 0.0027 | 0.0002 | 14.39 | 0.0024 | 0.0002 | 14.16 |  |
| β1 | -2.471 | 0.024 | -102.83 | -2.731 | 0.030 | -92.06 |  |
| β2 | -0.046 | 0.001 | -36.08 | -0.047 | 0.002 | -29.73 |  |
| β5 | 4.849 | 0.574 | 8.45 | 5.133 | 0.677 | 7.59 |  |
| β6 | 3.536 | 0.015 | 242.20 | 3.532 | 0.016 | 215.76 |  |
| β7 | 0.123 | 0.007 | 16.59 | 0.126 | 0.009 | 14.10 |  |
| β8 | 16.382 | 1.127 | 14.53 | 16.776 | 1.403 | 11.96 |  |
| β9 | 3.815 | 0.007 | 510.80 | 3.819 | 0.009 | 411.13 |  |
| β10 | (0.123) |  |  | (0.126) |  |  |  |
| β11 | 12.552 | 1.715 | 7.32 | 9.125 | 2.066 | 4.42 |  |
| β12 | 4.056 | 0.005 | 819.35 | 4.054 | 0.008 | 480.31 |  |
| β13 | 0.034 | 0.006 | 6.16 | 0.034 | 0.009 | 3.63 |  |
| parameter | estimate | SE | t-value | difference | SE | t-value | p-value^1^ |
| α | 0.0027 | 0.0002 | 14.39 | -0.0003 | 0.0003 | -1.20 | 0.232 |
| β1 | -2.471 | 0.024 | -102.83 | -0.260 | 0.038 | -6.80 | <0.001 |
| β2 | -0.046 | 0.001 | -36.08 | -0.001 | 0.002 | -0.38 | 0.702 |
| β5 | 4.849 | 0.574 | 8.45 | 0.284 | 0.887 | 0.32 | 0.750 |
| β6 | 3.536 | 0.015 | 242.20 | -0.004 | 0.022 | -0.19 | 0.853 |
| β7 | 0.123 | 0.007 | 16.59 | 0.003 | 0.012 | 0.24 | 0.813 |
| β8 | 16.382 | 1.127 | 14.53 | 0.394 | 1.800 | 0.22 | 0.827 |
| β9 | 3.815 | 0.007 | 510.80 | 0.003 | 0.012 | 0.29 | 0.771 |
| β10 | (0.123) |  |  | (0.003) |  |  |  |
| β11 | 12.552 | 1.715 | 7.32 | -3.427 | 2.685 | -1.28 | 0.204 |
| β12 | 4.056 | 0.005 | 819.35 | -0.003 | 0.010 | -0.28 | 0.779 |
| β13 | 0.034 | 0.006 | 6.16 | 0.000 | 0.011 | 0.00 | 0.997 |

Deviance= 334.0 (df=116), ^1^p-value for difference between estimates for males and females


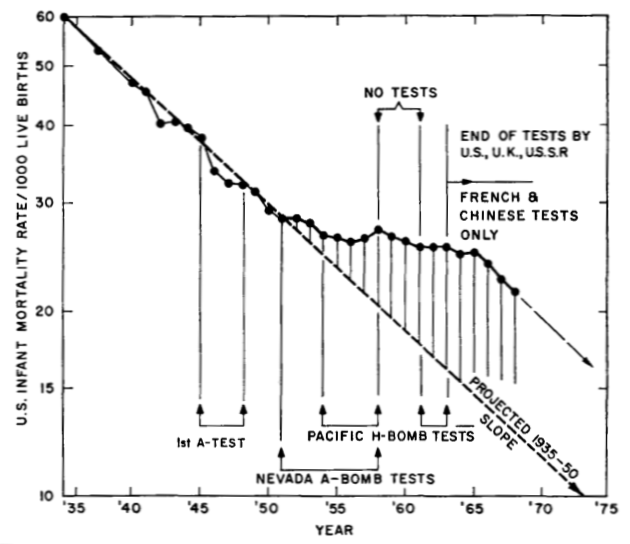


S1 Figure. Infant mortality rates in the U.S., 1935-1968
semi-log plot, with regression line and projected trend.
Data points in 1935, 1936, 1938, and 1939 are missing. From: Sternglass (1969), Figure 3


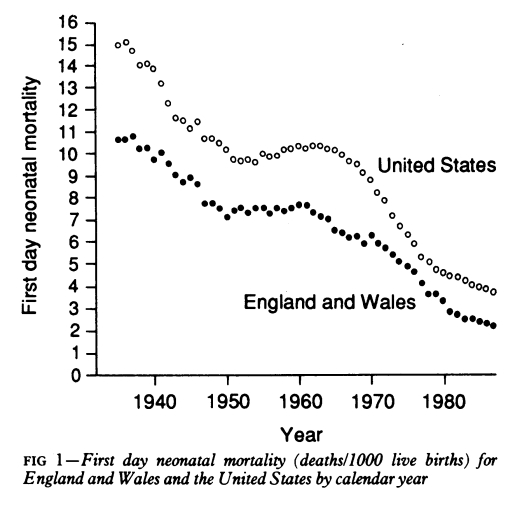


S2 Figure. First-day neonatal mortality in the United States and England and Wales
From (Whyte 1992), Fig 1


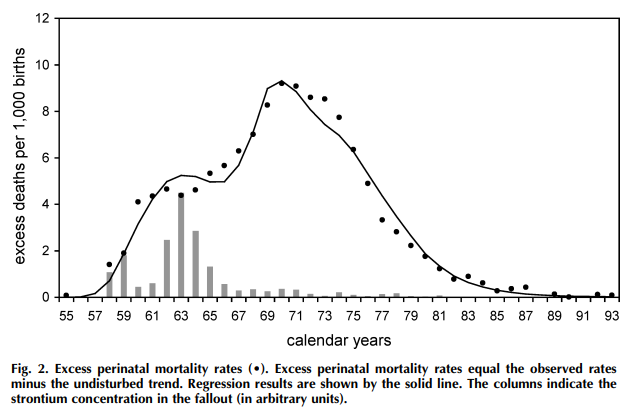


S3 Figure. Excess perinatal mortality rates in West Germany (former FRG), 1955-1993
From (Körblein 2004)


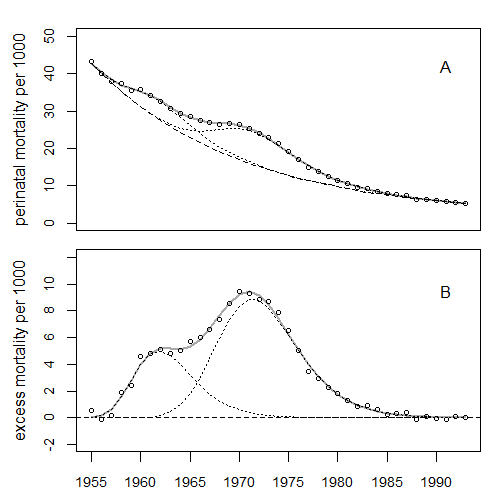


S4 Figure. Perinatal mortality in West Germany (former FRG), 1955-1993
and results of regression with two lognormal distributions superimposed on a uniformly declining trend. Panel B: Excess perinatal mortality rates and bell-shaped excess terms (thin dotted lines).


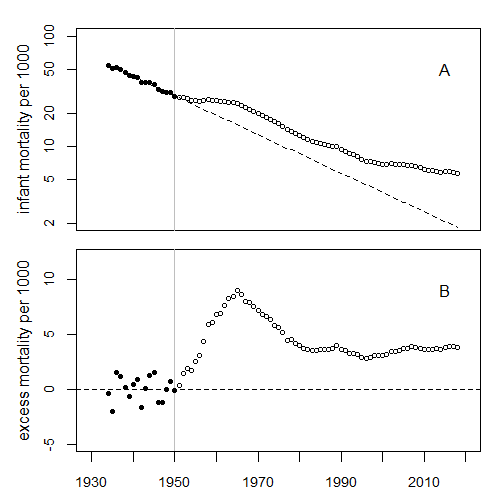


S5 Figure. Infant mortality rates in the U.S., 1934-2018, exponential trend
The dashed line shows the extrapolation of the exponential trend of the data in 1934-1950 (black dots).


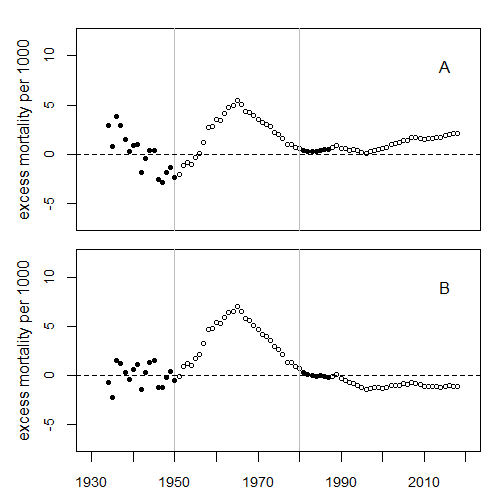


S6 Figure. Excess infant mortality rates in the U.S., 1934-2018
resulting from Poisson regression of the data in 1934-1950 and 1981-1987 with a linear (panel A) and a linear-quadratic (panel B) trend model.


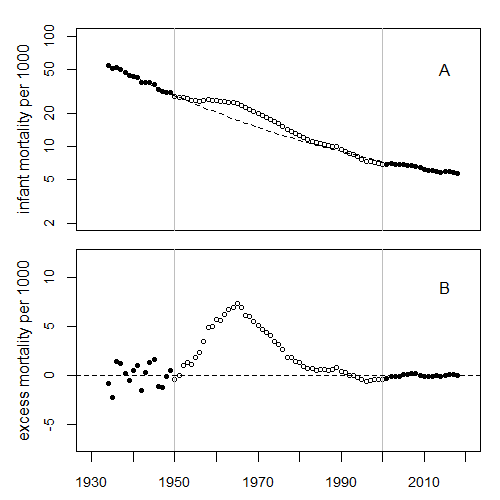


S7 Figure. Infant mortality rates in the U.S., 1934-2018
and result of Poisson regression without the 1950-2000 data (open circles) using a linear-quadratic temporal trend. Panel B: Excess infant mortality rates, i.e. observed rates minus rates predicted from regression without 1950-2000 data.


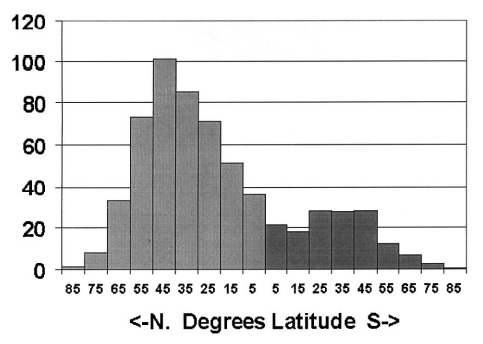


S8 Figure. Variation of total Sr-90 deposition with latitude (from [16)


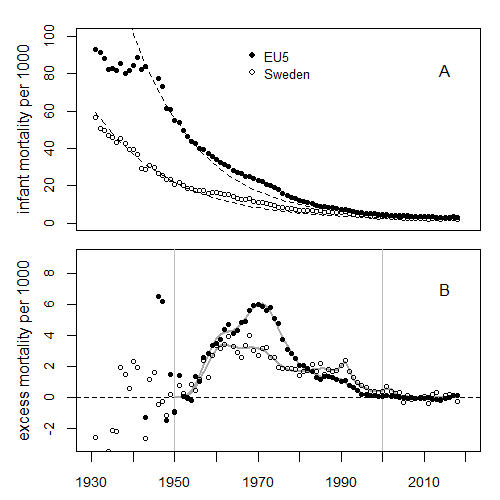


S9 Figure. Infant mortality rates in EU5 and Sweden
Panel B. Excess infant mortality rates and regression lines.


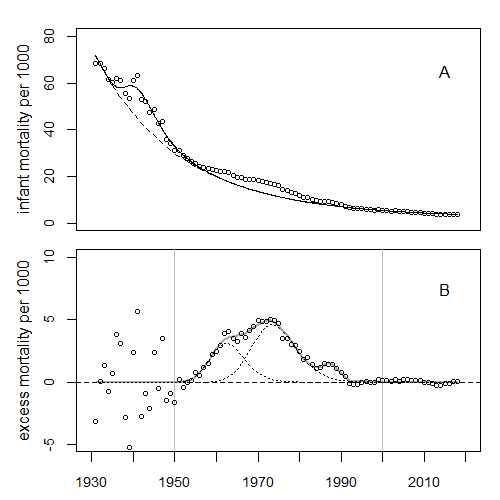

S10 Figure. Infant mortality in the United Kingdom, 1931-2018
Panel B: Excess infant mortality rates and lognormal distributions.

Lower panel: Deviations between observed and predicted (broken line in panel A) infant mortality rates, in units of standard deviations (standardized residuals).


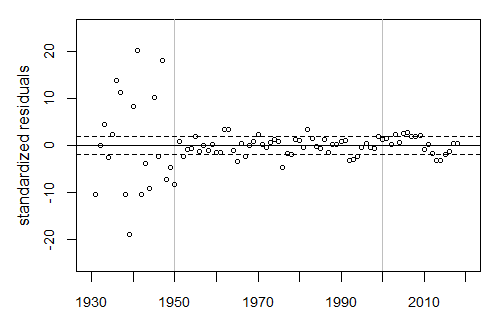


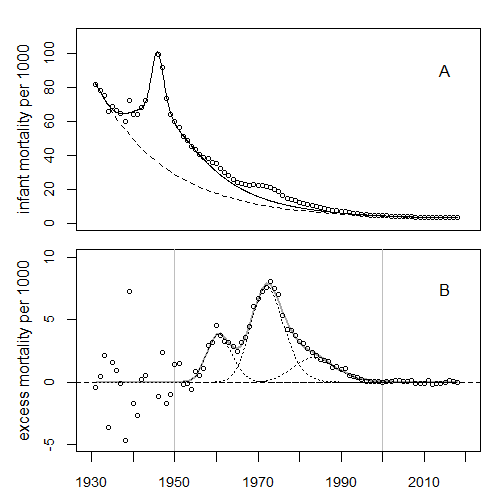


S11 Figure. Infant mortality in Germany, 1931-2018.
Data for 1944-1945 are not available. Panel B: Excess infant mortality rates and lognormal distributions.

Lower panel: Standardized residuals.


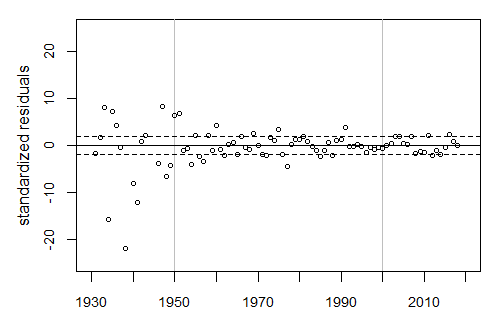


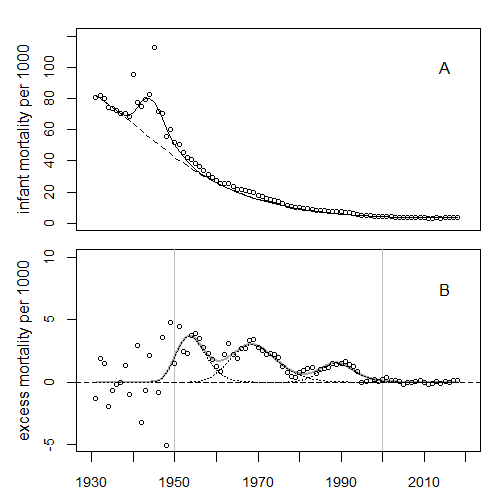


S12 Figure. Infant mortality in France, 1931-2018
Panel B: Excess infant mortality rates and lognormal distributions.

Lower panel: Standardized residuals.


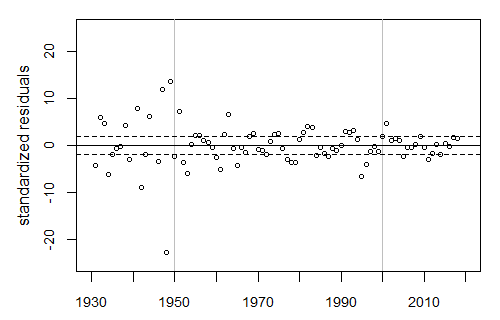


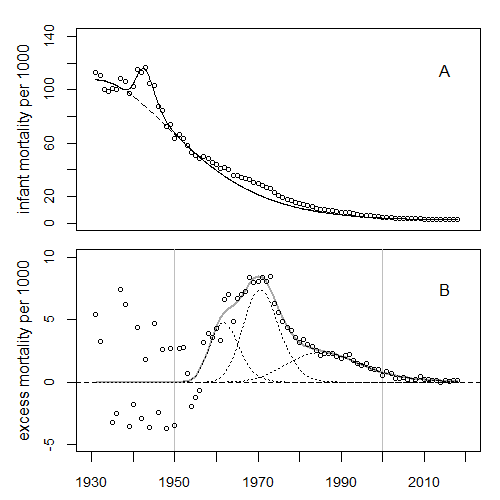


S13 Figure. Infant mortality in Italy, 1931-2018
and the result of regression with two lognormal distributions. Panel B: Excess infant mortality rates.

Lower panel: standardized residuals.


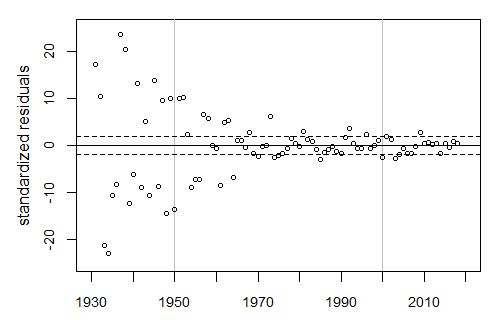


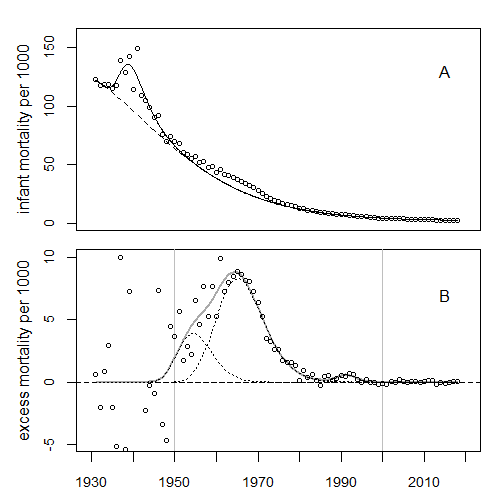


S14 Figure. Infant mortality in Spain, 1931-2018
and excess infant mortality rates (panel B)

Lower panel: standardized residuals.


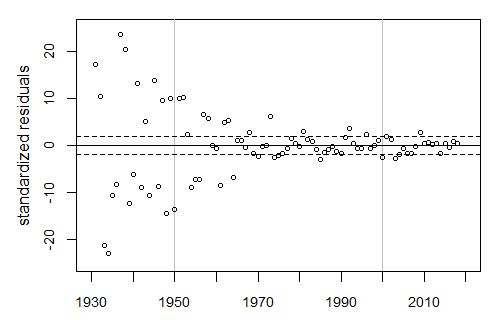


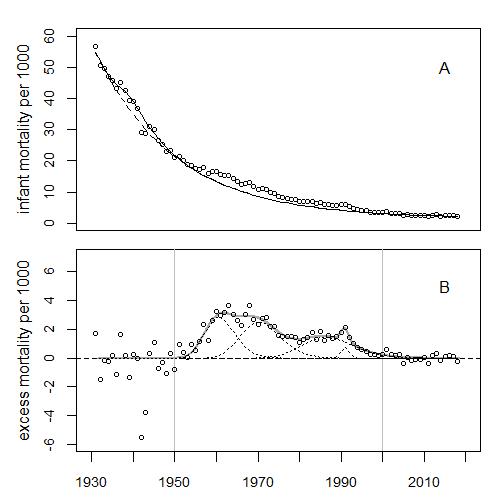


S15 Figure. Infant mortality in Sweden, 1931-2018
Outliers in 1942 and 1943 were omitted from the regression. Panel B: Excess infant mortality rates.

Lower panel: standardized residuals.


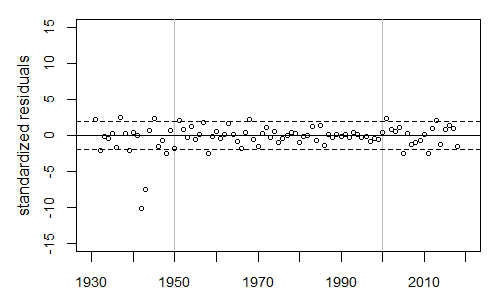


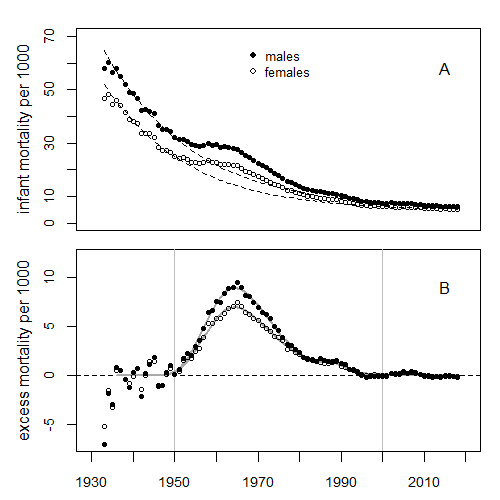


S16 Figure. Panel A: Infant mortality in the United States by gender, 1933-2018
Panel B: Excess infant mortality rates

Lower panel: standardized residuals


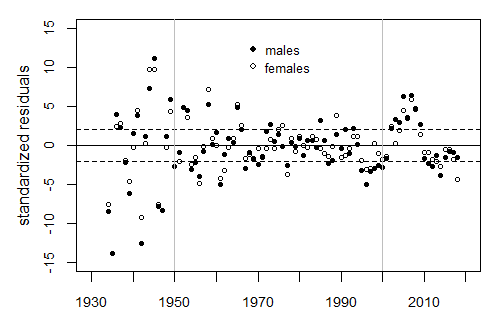


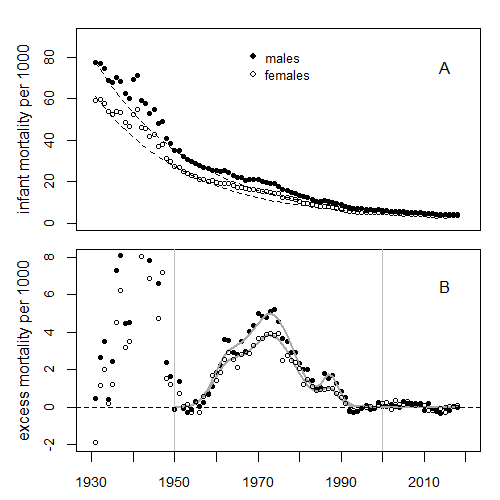


S17 Figure. Infant mortality in the United Kingdom by gender, 1931-2018
Panel B: Excess infant mortality rates.

Lower panel: standardized residuals.


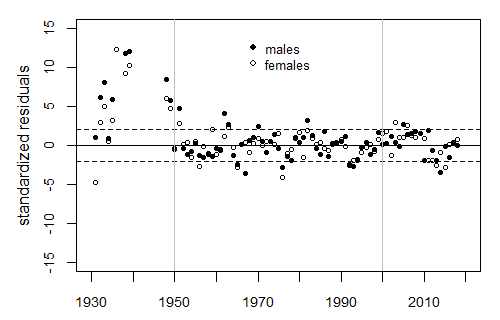


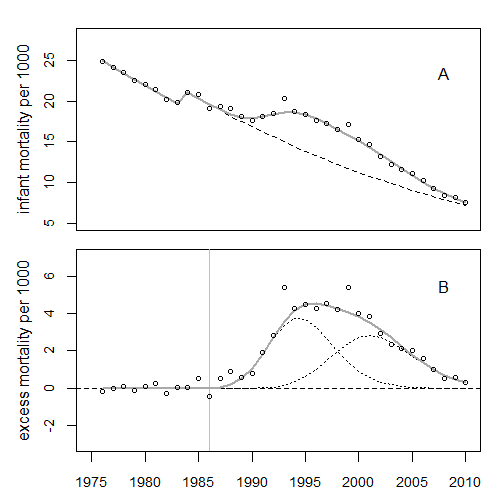


S18 Figure. Infant mortality in the Russian Federation, 1976-2010
Outliers in 1993 and 1999 were omitted from the regression. Panel B: Excess infant mortality rates and regression lines. The vertical line indicates 1986, the year of the Chernobyl accident.

Lower panel: standardized residuals.


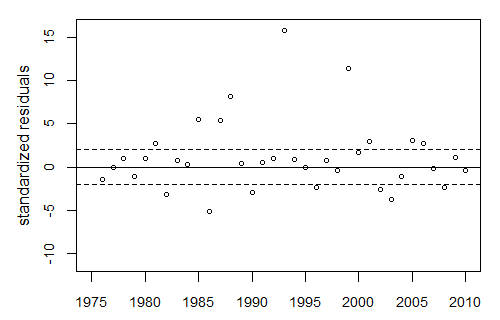

Supplement: S1 File — (DOCX) [file pone.0284482.s001.docx]
